# Heterologous Two-Dose Vaccination with Simian Adenovirus and Poxvirus Vectors Elicits Long-Lasting Cellular Immunity to Influenza Virus A in Healthy Adults

**DOI:** 10.1016/j.ebiom.2018.02.011

**Published:** 2018-02-15

**Authors:** L. Coughlan, S. Sridhar, R. Payne, M. Edmans, A. Milicic, N. Venkatraman, B. Lugonja, L. Clifton, C. Qi, P.M. Folegatti, A.M. Lawrie, R. Roberts, H. de Graaf, P. Sukhtankar, S.N. Faust, D.J.M. Lewis, T. Lambe, AVS Hill, S.C. Gilbert

**Affiliations:** a Icahn School of Medicine at Mount Sinai, Department of Microbiology, Annenberg Building, Room 16.30, One Gustave Levy Place, New York 10029, United States; b Centre for Statistics in Medicine, NDORMS, University of Oxford, Botnar Research Centre, Windmill Road, Oxford OX3 7LD, UK; c NIHR Wellcome Trust Clinical Research Facility, University of Southampton, University Hospital Southampton NHS Foundation Trust, Southampton, UK; d Clinical Research Centre, University of Surrey, Guildford GU2 7AX, UK; e The Jenner Institute, University of Oxford, ORCRB, Roosevelt Drive, Oxford OX3 7DQ, UK; f Sanofi Pasteur, MARCY l’ETOILE, 69280, France

**Keywords:** Influenza, T-cell responses, Influenza vaccines, Viral vectors, Adults, Older adults

## Abstract

**Background:**

T-cell responses against highly conserved influenza antigens have been previously associated with protection. However, these immune responses are poorly maintained following recovery from influenza infection and are not boosted by inactivated influenza vaccines. We have previously demonstrated the safety and immunogenicity of two viral vectored vaccines, modified vaccinia virus Ankara (MVA) and the chimpanzee adenovirus ChAdOx1 expressing conserved influenza virus antigens, nucleoprotein (NP) and matrix protein-1 (M1). We now report on the safety and long-term immunogenicity of multiple combination regimes of these vaccines in young and older adults.

**Methods:**

We conducted a Phase I open-label, randomized, multi-center study in 49 subjects aged 18–46 years and 24 subjects aged 50 years or over. Following vaccination, adverse events were recorded and the kinetics of the T cell response determined at multiple time points for up to 18 months.

**Findings:**

Both vaccines were well tolerated. A two dose heterologous vaccination regimen significantly increased the magnitude of pre-existing T-cell responses to NP and M1 after both doses in young and older adults. The fold-increase and peak immune responses after a single MVA-NP + M1 vaccination was significantly higher compared to ChAdOx1 NP + M1. In a mixed regression model, T-cell responses over 18 months were significantly higher following the two dose vaccination regimen of MVA/ChAdOx1 NP + M1.

**Interpretation:**

A two dose heterologous vaccination regimen of MVA/ChAdOx1 NP + M1 was safe and immunogenic in young and older adults, offering a promising vaccination strategy for inducing long-term broadly cross-reactive protection against influenza A.

**Funding Source:**

Medical Research Council UK, NIHR BMRC Oxford.

## Highlights

• Heterologous prime-boost vaccination regimens of MVA/ChAdOx1 NP+M1 are safe and immunogenic in young and older adults
• All MVA/ChAdOx1 NP+M1 regimens tested significantly increased cross-reactive T-cells
• Responses were durable and were maintained 18 months after vaccination
• The fold-increase after a single MVA-NP+M1 vaccination was significantly higher compared to ChAdOx1 NP+M1

Current seasonal influenza vaccines induce antibody responses to external glycoproteins, which are highly susceptible to the accumulation of mutations within antigenic sites, allowing escape from serological immunity conferred by prior infection or vaccination. In this phase I clinical study, we present the results of using two replication-deficient viral vectors expressing conserved influenza A antigens in four different vaccination regimens, administered at intervals of either 8 weeks or one year. We found that vaccination was safe and boosted T cell responses to influenza antigens substantially, with a further increase after the second vaccination, both in young and older adults.

## 1 Introduction

Influenza A virus (IAV) remains a significant global health problem causing seasonal epidemics and occasional pandemics. Vaccination is the most cost-effective public health intervention to combat influenza (Petrie et al., 2015). Current seasonal influenza vaccines induce humoral immune responses to external glycoproteins, hemagglutinin (HA) and neuraminidase (NA). However, the error-prone nature of influenza virus replication leads to the accumulation of drift mutations within antigenic sites, allowing escape from serological immunity conferred by prior infection or vaccination. The requirement to make advance predictions of which viruses to include in vaccines for the forthcoming influenza season can result in vaccine mismatches (Pebody et al., 2015). Additionally, responses to seasonal influenza vaccines are subtype-specific, only inducing immune responses to strains included in the vaccine and offer no heterosubtypic protection against novel subtype reassortants or emerging viruses like H5N1 or H7N9 avian influenza. This is particularly critical in the elderly in whom vaccine efficacy is lower, increasing their risk for severe illness (Jefferson et al., 2005; Haq and McElhaney, 2014; Rivetti et al., 2006).

A protective role for CD4^+^ and CD8^+^ T-cells in humans has been demonstrated in experimental challenge studies (McMichael et al., 1983b; Wilkinson et al., 2012). More recently, in community cohort studies, T-cells have been shown to be associated with reduced viral shedding and limited severity of illness. Sridhar et al. identified a correlation between the frequency of IFN-γ^+^/IL-2^−^ CD8^+^ T-cells and protection against symptomatic influenza (Sridhar et al., 2013). Hayward and colleagues found that higher frequencies of nucleoprotein (NP)-specific IFN-γ^+^ CD3^+^ T-cells were associated with a lower risk of symptomatic, PCR-confirmed influenza infection and viral shedding (Hayward et al., 2015). As the induction of such cross-protective T-cells following vaccination with current influenza vaccines is limited (He et al., 2006), alternative vaccination approaches to induce T-cell responses against highly conserved internal influenza antigens capable of protecting against antigenically distinct viruses with pandemic potential, such as NP or matrix protein 1 (M1), are needed. This would particularly benefit high-risk populations, such as the elderly, in whom there is a high risk of severe disease.

We have developed viral vectored vaccines, using the replication-deficient chimpanzee adenovirus ChAdOx1 and the attenuated orthopoxvirus modified vaccinia virus Ankara (MVA) expressing NP and M1 influenza virus antigens (ChAdOx1 NP + M1 and MVA-NP + M1) as one approach to combat this problem. We have previously demonstrated that a single dose of these viral vector vaccines is safe and immunogenic (Antrobus et al., 2012; Lillie et al., 2012; Antrobus et al., 2014b) and in a proof-of-concept experimental influenza challenge study, showed that vaccination with MVA-NP + M1 can reduce the duration of viral shedding (Lillie et al., 2012). However, whether a vaccination strategy using a heterologous combination of these two viral vectors is synergistic in inducing higher magnitude, improved quality and longer durability of T-cell responses, as seen with other antigens, is not known. We conducted this randomized, open-label, Phase I clinical trial to assess the safety and cellular immunogenicity of prime/boost vaccination regimes employing MVA-NP + M1 and ChAdOx1 NP + M1 in young and older adults.

## 2 Materials and Methods

### 2.1 ChAdOx1 NP + M1 and MVA-NP + M1 Vaccines

Both vaccines have been described previously and consist of viral vectors expressing NP and M1 antigens from influenza A virus (H3N2, A/Panama/2007/99) as a single fusion protein (Antrobus et al., 2014b; Berthoud et al., 2011). MVA-NP + M1 was administered at a dose of 1·5 × 10^8^ plaque forming units (pfu) in 1·15 ml while ChAdOx1 NP + M1 was administered at a dose of 2·5 × 10^10^ viral particles (vp) in 0·22 ml.

### 2.2 Study Design and Participants

**Fig. 1:**
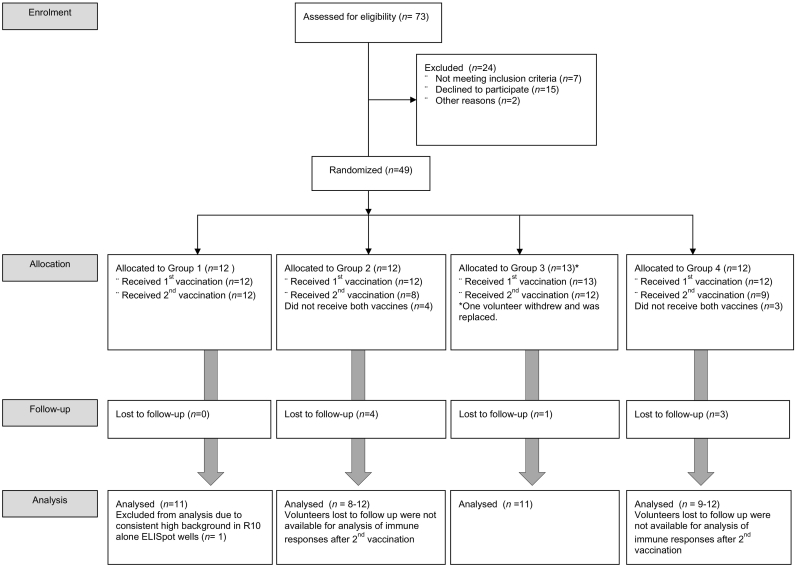
CONSORT flow diagram of the trial (Groups 1–4). Forty-nine subjects aged 18–46 years were enrolled into four groups (G1–4) with 12 participants in each group. One volunteer in group 3 withdrew soon after enrolment.

**Fig. 2:**
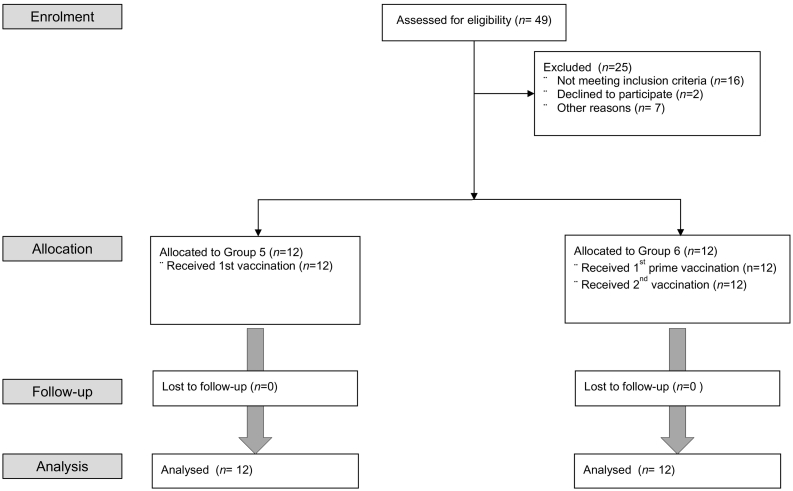
CONSORT flow diagram of the trial (Groups 5&6). A further twenty-four subjects aged ≥50 years were enrolled into two additional groups, G5 and G6 (12/group).

The study was a Phase I open-label, randomized, multi-center study conducted at the Centre for Clinical Vaccinology and Tropical Medicine, Oxford, UK, Surrey Clinical Research Centre, University of Surrey, UK and NIHR Wellcome Trust Clinical Research Facility, Southampton, UK (Table 1) (CONSORT diagram: Fig. 1, Fig. 2). Healthy adults aged 18–46 (Groups 1–4) and 50 years or over (Groups 5 and 6) (Table 1) were eligible to participate in the trial after providing written informed consent. Full details of eligibility criteria are described in the trial protocol provided in the Supplementary material. All volunteers were healthy adults with negative pre-vaccination tests for HIV antibodies, hepatitis B surface antigen, hepatitis C antibodies and urine pregnancy test. Written informed consent was obtained in all cases and the study was conducted in accordance with the principles of the Declaration of Helsinki. For Groups 1–4, participants were randomized in variable block sizes according to vaccine allocation (ChAdOx1 NP + M1 or MVA-NP + M1 as the first vaccine) but not according to interval duration, which was determined by the preference of the volunteer until groups were full. The same randomization method was used to randomize participants to group 5 (ChAdOx1 NP + M1 only), or 6 (ChAdOx1 NP + M1 followed by MVA-NP + M1 8 weeks later). This was an open label study with subjects and investigators unblinded to the allocated group but study personnel conducting the immunology assays were blinded to group allocation. The clinical trial was approved within the UK by the regulatory authority (reference 21,584/0311/001-0001) and the Oxfordshire National Research Ethics Service Committee (OXREC A 13/SC/0004). The trial is registered at www.clinicaltrials.gov (Identifier: NCT01818362).

**Table 1:** Trial study design and participant demographics.

Groups	Number of participants	Average age in years (SD, range)	Sex of participants (Number/%)	1st vaccination W0	2nd vaccination W8	2nd vaccination W52	Follow up post-immunization (weeks)
Group 1	12	25·5 (7·4, 21–45)	M: 3 (25%) F: 9 (75%)	ChAdOx1 NP + M1	MVA-NP + M1	–	W1, W2, W3, W4, W8, W9, W10, W11, W26, W52, W78
Group 2	12	24·8 (6·6, 19–39)	M: 5 (41·7%) F: 7 (58·3%)	ChAdOx1 NP + M1	–	MVA-NP + M1	W1, W2, W3, W4, W8, W26, W52, W53, W54, W55, W78
Group 3	13^a^	24·1 (5·3 20–41)	M: 5 (38·5%) F: 8 (61·5%)	MVA-NP + M1	ChAdOx1 NP + M1	–	W1, W2, W3, W4, W8, W9, W10, W11, W26, W52, W78
Group 4	12	25·6 (7·2, 20–46)	M: 3 (25%) F: 9 (75%)	MVA-NP + M1	–	ChAdOx1 NP + M1	W1, W2, W3, W4, W8, W26, W52, W53, W54, W55, W78
Group 5	12	61·4 (6·0, 52–72)	M: 4 (33·3%) F: 8 (66·6%)	ChAdOx1 NP + M1	–	–	W1, W2, W4, W8, W26
Group 6	12	61·6 (8·4, 50–78)	M: 5 (41·7%) F: 7 (58·3%)	ChAdOx1 NP + M1	MVA-NP + M1	–	W1, W2, W4, W8, W9, W12 W26

a One individual in Group 3 withdrew early after first vaccination and was replaced.

### 2.3 Study Procedures

All volunteers in Groups 1–4 were vaccinated on the day of enrolment and either 8 or 52 weeks later (CONSORT diagram: Fig. 1, Fig. 2). Volunteers in Group 5 were vaccinated with a single dose of ChAdOx1 NP + M1 on the day of enrolment and volunteers in Group 6 were vaccinated with ChAdOx1 NP + M1 on the day of enrolment followed 8 weeks later by MVA-NP + M1. All vaccines were administered by an intramuscular (*im*) injection into the deltoid region of the arm. Volunteers were reviewed in clinic 24 h after vaccination for potential adverse events (AE) and were provided with a diary card to record solicited and unsolicited AEs which was reviewed at follow-up visits. Blood samples for safety and immunogenicity were collected at each follow-up visit (see Table 1 for timings). Safety events were assessed as the occurrence of local and systemic reactogenicity signs and symptoms for 7 days following vaccination procedures. Occurrences of serious adverse events were assessed during the whole study duration and changes from baseline were used for safety laboratory measures. Interferon gamma Enzyme-Linked ImmunoSpot assays (ELISpots) were used as a marker of cell mediated response at baseline and different time points throughout the trial.

### 2.4 Statistical Analysis

As a Phase I study with no predefined hypotheses, formal power calculations were not performed. With 12 subjects per group (Group 1–4), it was estimated that there would be 88% power (alpha = 0·05) to observe a three-fold increase in T-cell response to NP and M1 pre-vaccination to peak levels post-vaccination, although this was not the primary endpoint. With 10 per group, we would have 80% power and 68% power with 8 per group. This informal power calculation was carried out based on immune responses obtained from our previous trials with single use of ChAdOx1 NP + M1 and MVA-NP + M1.

All participants were included in safety analysis with safety data presented according to frequency, severity and duration of adverse events. The primary immunogenicity analysis compared the area under the curve (AUC) of the T-cell response (IFN-γ SFC/million peripheral blood mononuclear cells [PBMCs]) from baseline to week 78 for Groups 1 + 2 vs 3 + 4, or from baseline to week 26 for Group 5 vs 6. For the primary analysis, a *t*-test was performed on the intention to treat (ITT) population. The AUC was calculated using the trapezium method. Where a response at a time point was missing, we took the mean of imputed values from twenty imputed datasets (generated using multiple imputation by chained equations and the predictive mean matching method). Secondary analyses were performed using a t-test on the available data only, to compare between pre-specific groups. No formal adjustment for multiple significance testing was carried out for this phase I study, but note that all other analyses were exploratory. Primary, secondary and post-hoc immunogenicity analyses were carried out using STATA 14.2: StataCorp. 2015. *Stata Statistical Software: Release 14*. College Station, TX: StataCorp LP.

Exploratory immunogenicity data were analyzed using GraphPad Prism version 5.04 for Windows (GraphPad Software Inc., California, USA) and non-parametric analyses. To compare ELISpot responses between selected, matched time-points in a group, a Wilcoxon matched-pairs signed rank test was used. To compare baseline IFN-γ ELISpot responses between G1-6 a 1-way ANOVA was used with Kruskal-Wallis test and Dunn's multiple comparison test. Area under the curve (AUC) analysis can be used in immunological studies as a tool to estimate overall vaccine immunogenicity and durability (Heldens et al., 2002). AUC values for identical time periods post-vaccination were calculated per individual for each time point. AUC is presented as AUC/total number of days post-vaccination when comparing between groups which did not have match volunteer visits. In order to calculate AUC values when timepoints were missing due to absent volunteer visits or ELISpot plate failures, the average of the group at that time-point was used for analysis. The resulting data sets were analyzed using a non-parametric two-tailed *t*-test (Mann-Whitney).

### 2.5 Ex Vivo IFN-γ ELISpot

Ex vivo interferon-gamma enzyme-linked immunosorbent spot (IFN-γ ELISpot) assays were performed using fresh PBMC to determine responses to the NP + M1 vaccine antigen at each timepoint (Antrobus et al., 2014b). The breadth of the NP + M1-specific T-cell response was determined using 8 peptide pools, each pool containing ten 15-20mer peptides overlapping by 10 amino acids, spanning the complete NP + M1 insert.

### 2.6 Intracellular Cytokine Staining (ICS) and Analysis by Flow Cytometry

Cryopreserved PBMCs were incubated with co-stimulatory antibodies αCD28 and αCD49d and anti-CD107a. Further details of the staining procedure are outlined in the *Supplementary methods* and the details of antibodies used presented in Supplementary Table.1. Briefly, monocytes (CD14^+^), B-cells (CD19^+^) and NK cells (CD56^+^) were excluded from analysis. Cells were gated on lymphocytes, singlets, live cells, CD3^+^CD14^−^CD19^−^, CD4^+^CD8^−^ or CD8^+^CD4^−^, and then assessed for IFN-γ, IL-2, TNFα secretion and combinations of these cytokines.

## 3 Results

### 3.1 Study Population

A total of 49 healthy volunteers aged 18–46 were enrolled and randomized into Groups 1–4 (G1–4) to assess the safety and immunogenicity of a heterologous two dose regimen of ChAdOx1 NP + M1 and MVA-NP + M1. The average age of the 49 participants who received at least one vaccination was 25 years (SD: 6·47, range: 19–46 years) and 16/49 (32%) participants were male. One volunteer in G3 withdrew within the first week after receiving the first MVA-NP + M1 vaccination and was replaced. Eight further subjects withdrew from the study, 4 after receiving ChAdOx1 NP + M1 in G2 prior to receiving the second vaccination, 3 in G4 prior to receiving the second vaccination and 1 in G3 after receiving both vaccinations. In Group 5 and Group 6 (G5 and G6), 24 healthy volunteers aged 50 or over were enrolled. The average age of the 24 participants was 61.5 years (SD: 7.14, range: 50–78 years) and 9/24 (37.5%) were male.

### 3.2 Vaccine Safety

Administration of ChAdOx1 NP + M1 and MVA-NP + M1 vaccines was found to be safe and well-tolerated, in agreement with our previous studies.(Antrobus et al., 2014a; Berthoud et al., 2011; Lillie et al., 2012; Antrobus et al., 2014b; Antrobus et al., 2012) No vaccine-related serious AEs were observed over the duration of the study. The majority of local and systemic AEs in all groups were mild to moderate in nature and resolved spontaneously within 1–2 days (Table.2). The proportion of participants experiencing local and systemic AEs of any severity after ChAdOx1 NP + M1 or MVA-NP + M1 vaccination was significantly lower (*p* < 0.01) in individuals ≥ 50 years compared to individuals 18–46 years.

**Table 2:** Vaccine safety and reactogenicity.

	Severity of AEs	Local arm pain	Redness	Swelling	Warmth	Itch	Documented fever	Feverishness	Arthralgia	Myalgia	Fatigue	Headache	Nausea	Malaise
ChAd prime G1 and G2	Mild	13	4	11	3	0	3	2	3	8	8	10	4	4
	Moderate	9	0	0	0	0	0	7	4	6	4	3	2	5
	Severe	0	0	0	0	0	0	3	1	1	2	2	0	1
	Any severity	22	4	11	3	0	3	12	8	15	14	15	6	10
MVA prime G3 and G4	Mild	11	3	10	6	0	0	7	4	7	12	10	7	13
	Moderate	11	0	0	0	0	0	3	1	6	3	5	3	1
	Severe	2	0	0	0	0	0	0	1	1	1	1	0	1
	Any severity	24	3	10	6	0	0	10	6	14	16	16	10	15
ChAd prime G5 and G6 (≥50 years)	Mild	10	2	4	1	1	1	2	4	4	8	4	3	3
	Moderate	2	0	0	0	1	1	3	3	3	2	9	0	0
	Severe	0	0	0	0	0	0	0	0	0	0	0	0	0
	Any severity	12	2	4	1	2	2	5	7	7	10	13	3	3
MVA boost at 8 weeks G1	Mild	5	1	5	3	0	1	4	3	4	4	5	2	4
	Moderate	5	0	1	0	0	0	3	1	1	2	2	1	4
	Severe	2	0	1	0	0	**0**	0	2	2	1	0	0	0
	Any severity	12	1	7	3	0	1	7	6	7	7	7	3	8
MVA boost at 52 weeks G2	Mild	6	2	5	3	0	0	5	3	6	6	6	1	6
	Moderate	2	0	0	0	0	**0**	0	0	0	0	1	1	0
	Severe	0	0	0	0	0	0	0	0	0	0	0	0	0
	Any severity	8	2	5	3	0	0	5	3	6	6	7	2	6
ChAd boost at 8 weeks G3	Mild	9	3	4	4	1	**0**	3	2	4	4	4	3	4
	Moderate	1	0	0	0	0	1	2	0	0	1	3	0	1
	Severe	0	0	0	0	0	0	0	0	0	0	0	1	0
	Any severity	10	3	4	4	1	1	5	2	4	5	7	4	5
ChAd boost at 52 weeks G4	Mild	5	2	2	3	1	2	3	2	3	4	0	2	1
	Moderate	3	0	0	1	0	1	0	3	4	3	0	0	3
	Severe	1	0	0	0	0	0	2	0	0	0	0	0	0
	Any severity	9	2	2	4	1	3	5	5	7	7	0	2	4
MVA boost at 52 weeks G6 (≥50 years)	Mild	3	0	1	1	0	0	0	1	2	3	1	1	2
	Moderate	5	0	0	0	0	0	0	2	3	0	0	0	1
	Severe	1	0	0	0	0	0	0	0	0	0	0	0	0
	Any severity	9	0	1	1	0	0	0	3	5	3	1	1	3

### 3.3 Vaccine Immunogenicity in Young Adults

**Fig. 3:**
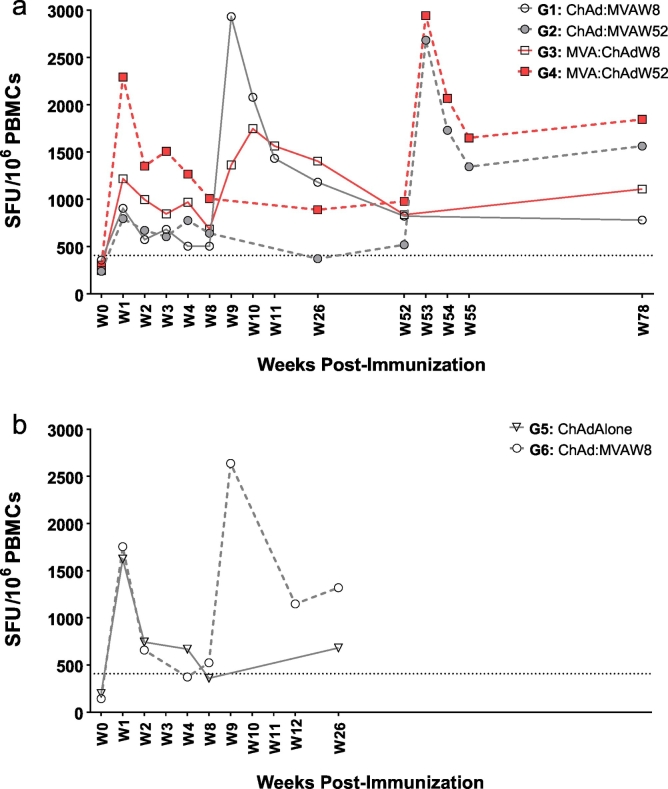
Ex vivo IFN-γ median ELISpot responses to influenza antigen NP + M1 in volunteers aged 18–46 and 50+ following vaccination with viral vectored vaccines. (a) G1-4: Median ELISPOT Responses (NP + M1); (b) G5&6: Median ELISPOT Responses (NP + M1). (a) Median IFN-γ ELISpot responses from vaccinated volunteers at baseline (W0) and at intervals following first (V1) and second vaccinations (V2) for Groups 1–4. Volunteers were first vaccinated intramuscularly (*im*) with ChAdOx1 NP + M1 (2.5 × 10^10^vp) and subsequently vaccinated with MVA-NP + M1 (1.5 × 10^8^ pfu) at week 8 (*G1:* W8) or week 52 (*G2:* W52). Alternatively, volunteers were vaccinated *im* with MVA-NP + M1 (1.5 × 10^8^ pfu) followed by vaccination with ChAdOx1 NP + M1 at W8 (*G3*) or W52 (*G4*). (b) Volunteers aged 50+ were vaccinated *im* with a single dose (*G5*) of ChAdOx1 NP + M1 (2.5 × 10^10^vp) or vaccinated with ChAdOx1 NP + M1 followed with MVA-NP + M1 (1·5 × 10^8^ PFU) at W8 (*G6*). PBMC were stimulated with overlapping pools of peptides corresponding to the NP + M1 vaccine antigen. Controls included cells stimulated with PHA/SEB, PPD or irrelevant peptide TRAP33 (*data not shown*). Negative control was cells stimulated with media alone (*data not shown*). Differences in responses between selected time-points were determined using a two-tailed *t*-test using Wilcoxon signed-rank test for matched pairs. *P < 0.05, **P < 0.01, ***P < 0.001, NS = *P* > 0.05.

The primary immunogenicity endpoint was the frequency of antigen-specific T-cells measured by IFN-γ ELISpot (Fig. 3a). There was no significant difference in frequency of pre-existing NP + M1 peptide-specific T-cells prior to vaccination between groups and the median response was consistent with responses measured in our previous clinical trial (Antrobus et al., 2014b) (Supplementary Fig. 1). The primary analysis compared ChAdOx1/MVA (combining G1 and G2) and MVA/ChAdOx1 (G3 and G4 combined) vaccination regimes. There was no significant difference in the AUC over the entire trial duration of 72 weeks between ChAdOx1/MVA (G1 + G2) and MVA/ChAdOx1 (G3 + G4) although mixed linear regression of all available responses from all time points adjusting for time point and baseline values revealed significantly higher responses (*p* = 0.007) with the MVA/ChAdOx1 regimen than ChadOx1/MVA regimen. Post-hoc analysis of the AUC of immune response showed no significant difference between those who received the second vaccine at week 8 and those who received the second vaccine at week 52 (i.e. Group 1 + 3 vs Group 2 + 4).

Regardless of vaccination regimen, T-cell responses measured at the last time point 18 months after the first vaccination were significantly higher than pre-existing baseline levels (G1, *p* *=* 0.0049; G2, *p* = 0.03; G3, *p* = 0.001; G4, *p* = 0.04) (Fig. 3a). Similarly, even 52 weeks after a single vaccination with ChAdOx1 NP + M1 (G2) or MVA-NP + M1 (G4), T-cell responses were maintained at levels significantly higher than baseline in both groups (G2: 520 SFU/10^6^ PBMC, *p* = 0.02; G4: 978·3 SFU/10^6^ PBMC, *p* = 0.02).

MVA-NP + M1 and ChAdOx1 NP + M1 boosted T-cell responses to significantly higher frequencies either as the first or second vaccination with peak responses typically observed one week after vaccination (Fig. 3a). In G1 and G2, ChAdOx1 NP + M1 vaccination was followed by MVA-NP + M1 8 weeks (W8) or 52 weeks (W52) later, respectively with peak responses ~5.8-fold higher (2932 SFU/10^6^ PBMC; *p* = 0.004) in G1 and ~5-fold higher (2683 SFU/10^6^ PBMC; *p* = 0.02) in G3 compared to responses prior to MVA-NP + M1 vaccination. In G3 and G4, MVA-NP + M1 was followed by ChAdOx1 NP + M1 at W8 or W52, respectively. Peak responses following ChAdOx1 NP + M1 boost vaccination were elevated ~2-fold (1364 SFU/10^6^ PBMC; *p* = 0.03) in G3 and ~3-fold (2942 SFU/10^6^ PBMC, *p* = 0.02) compared to responses prior to ChAdOx1 NP + M1 boost (Fig. 3a).

**Fig. 4:**
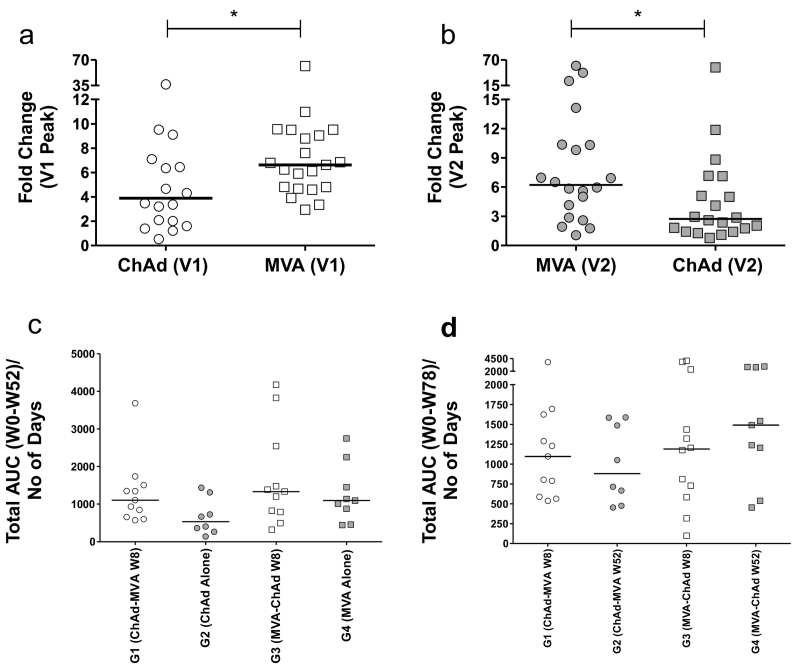
Comparison of immune responses following 1st (V1) and 2nd (V2) vaccination with ChAd or MVA viral vectors. (a) G1-G2 vs G3-G4; (b) G1-G2 vs G3-G4; (c) G1-G4; (d) G1-G4. (a) Comparison of fold changes in IFN-γ immunogenicity at the peak of the response following primary vaccination with either ChAdOx1 NP + M1 (G1&G2) or MVA-NP + M1 (G3&G4). (b) Comparison of fold changes in IFN-γ immunogenicity at the peak of the response following a second heterologous vaccination with MVA-NP + M1 (G1&G2) or ChAdOx1 NP + M1 (G3&G4). The fold-increase in peak T-cell response over baseline following the first (V1) MVA-NP + M1 vaccination was significantly higher (~6-fold increase over baseline *p* = 0.048, median = 251.7 SFU/10^6^ PBMC) than that induced by ChAdOx1 NP + M1 (~4-fold increase over baseline, median = 265 SFU/10^6^ PBMC). In comparing the fold-change in the peak of the response following the second vaccination (V2), we observed responses induced by MVA-NP + M1 were ~2·3-fold greater (*p* = 0.02) than that induced by ChAdOx1 NP + M1. (c) AUC analysis for each group in the 52 week interval was carried out to compare the effect of a second vaccination at W8 versus no V2 vaccination on overall immunogenicity. (d) AUC analysis comparing overall immunogenicity for each group for the duration of the study (W0-W78). Abbreviations are as follows; AUC = area under the curve, ChAd = ChAdOx1 NP + M1 and MVA = MVA-NP + M1. Line represents the median. Differences between groups were calculated using an unpaired non-parametric test (Mann-Whitney). * = *p* < 0.05.

We compared the fold-change in IFN-γ^+^ ELISpot response between peak and pre-vaccination frequencies after ChAdOx1 NP + M1 or MVA-NP + M1 vaccination (Fig. 4a, b). The fold-increase in pre-existing T-cell responses after MVA-NP + M1 was significantly higher than ChAdOx1 NP + M1, when administered as either the first (V1) or the second (V2) vaccination. In addition to the fold-increase, median peak T-cell responses after the first vaccination with MVA-NP + M1 (2023 SFCs/million PBMCs; LQ and UQ: 1349–2750) were significantly higher (*p* = 0.01) than ChAdOx1-NP + M1 (1147 SFCs/million PBMCs; LQ and UQ: 665–1953). No statistically significant difference was observed in peak responses after the second dose.

### 3.4 Vaccine Immunogenicity in Older Adults

Improving T-cell responses in older adults is a long standing goal for improved influenza vaccines and we evaluated the immunogenicity of the ChAdOx1 NP + M1 vector either as a single (G5) or a two dose regimen with MVA-NP + M1 (G6) in adults aged ≥50 years (Fig. 3b). In order to determine if the second vaccination with MVA-NP + M1 improved the overall response (W0-W26), we compared the AUC between G5 and G6 (Supplementary Fig. 2). The combination of vaccination with ChAdOx1 NP + M1 followed by MVA-NP + M1 had higher AUC of immune response compared to ChAdOx1 NP + M1 alone (*p* = 0.04), although this difference was not significant when an outlier was excluded from Group 6 (*p* = 0.08). Responses to NP + M1 were increased >8-fold (median 1623 SFU/10^6^ PBMC; *p* = 0.008) for G5 and ~12-fold (median 1755 SFU/10^6^ PBMC; *p* = 0.02) for G6 when compared with baseline responses of 198 SFU/10^6^ and 143 SFU/10^6^ respectively (Fig. 3b). The mean peak immune response after a single dose of ChAdOx1 NP + M1 was higher in older adults (Groups 5 + 6: median 2036 SFU/10^6^ PBMCs) compared to younger adults (Group 1 + 2: median 1147 SFU/10^6^ PBMCs).

Importantly, 6 months after the first vaccination, IFN-γ responses in both groups were maintained at levels ~2.5-fold (G5) and 9-fold (G6) higher than baseline.

### 3.5 Analysis of NP Specific IFN-γ ELISpot Responses

**Fig. 5:**
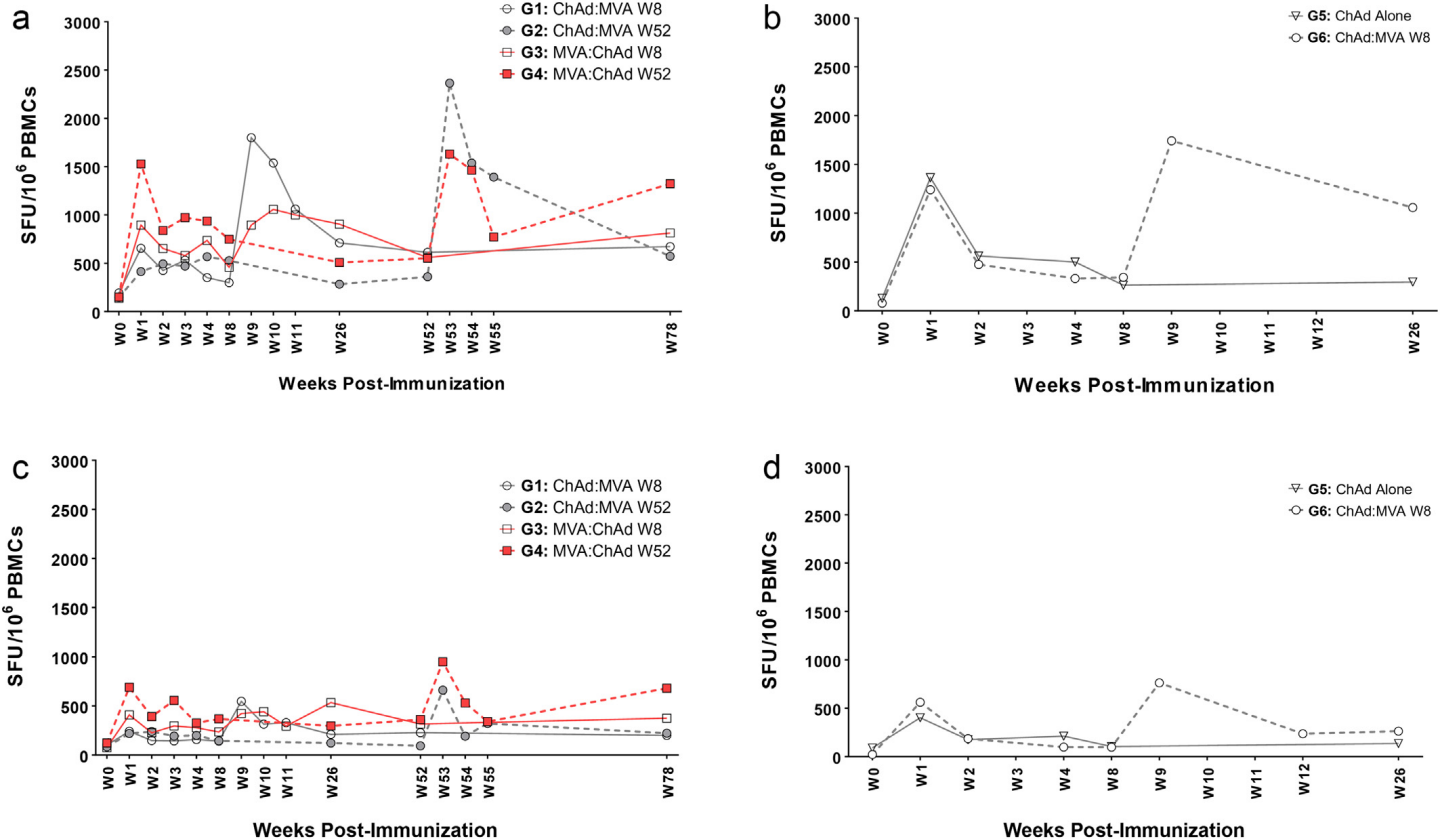
Separate median IFN-γ ELISpot responses for NP or M1 in vaccinated volunteers. (a) G1–4: NP Median ELISPOT Response; (b) G5&6: NP Median ELISPOT Response; (c) G1-4: M1 Median ELISPOT Response; (d) G5&6: M1 Median ELISPOT Response. Median IFN-γ ELISpot responses from healthy volunteers at baseline (W0) and at intervals following first and second vaccinations for NP (a, b) or M1 alone (c, d). Volunteers aged 18–46 (a, c) were vaccinated *im* with ChAdOx1 NP + M1 (2·5x10^10^vp) followed by a 2nd vaccination with MVA-NP + M1 (1·5 × 10^8^ pfu) at week 8 (W8; *G1*) or week 52 (W52: *G2*). Alternatively, volunteers were vaccinated IM with MVA-NP + M1 (1·5 × 10^8^ pfu) and then with ChAdOx1 NP + M1 at W8 (*G3*) or W52 (*G4*). Volunteers aged 50+ in *G5&6* (b, d) were vaccinated *im* with a single dose of ChAdOx1 NP + M1 (2.5 × 10^10^vp) or vaccinated with ChAdOx1 NP + M1 followed with MVA-NP + M1 (1·5 × 10^8^ PFU) at W8. PBMCs were stimulated with overlapping pools of peptides corresponding to the NP + M1 vaccine antigen. NP or M1 specific responses were calculated by subtracting responses for P6M1 peptides alone from the P6 response. NP responses are to P1–5 with P6 NP peptides alone. M1 responses are to P6 M1 epitopes with P7 and P8 responses. Controls included cells stimulated with PHA/SEB, PPD or irrelevant peptide TRAP33 (data not shown). Negative control was cells stimulated with media alone (*data not shown*). Differences in responses between selected time-points were determined using a two-tailed *t*-test using Wilcoxon signed-rank test for matched pairs. *P < 0.05, **P < 0.01, ***P < 0.001, NS = *P* > 0.05.

As a critical role for NP-specific T-cell responses in protection from symptomatic influenza and reducing viral shedding has recently been demonstrated (Hayward et al., 2015), we measured IFN-γ ELISpot responses specific for NP to determine if ChAdOx1 NP + M1 and MVA-NP + M1 vaccination boosted these cross-reactive T-cell responses to NP (Fig. 5a-d). In young adults (G1-G4), pre-existing baseline responses to NP were boosted to significantly higher levels following vaccination and durably maintained at levels significantly higher than baseline for 18 months after the first vaccination, regardless of the vaccination regimen employed (Fig. 5a, c). Responses to M1 antigen (253 amino acid residues) were also significantly boosted and maintained at levels significantly higher than baseline for 18 months after the first vaccination in all groups except G1.

In adults ≥ 50 years (G5 and G6), frequencies of NP or M1-specific T-cells 8 weeks and 26 weeks after a single dose of ChAdOx1 NP + M1 were not significantly higher than pre-vaccination levels (Fig. 5b, d). However, the two dose (G6) vaccination regimen induced responses to both NP and M1 at significantly higher frequencies (NP: *p* = 0.0068; M1: *p* = 0.0136) than pre-vaccination levels up to 6 months after first vaccination.

We also measured the breadth of the IAV-specific T-cell response to eight individual NP + M1 peptide pools by IFN-γ^+^ ELISpot (Supplementary Fig. 3). We detected broad antigen-specific T-cell responses across all peptide pools prior to vaccination (W0). Vaccination boosted pre-existing responses to all peptide pools but did not increase the breadth of the response by inducing responses to peptide pools in which there was no prior response.

### 3.6 Phenotype of IAV-Specific IFN-γ^+^ CD8^+^ T-Cells

A subset of samples were analyzed by flow cytometry to characterize the different T-cell memory subsets (Supplementary Table.1, Supplementary Fig. 4, Supplementary Fig. 5, Supplementary Fig. 6). Populations of IFN-γ^+^ CD8^+^ T-cells were functionally phenotyped as naïve cells (CD45RA^+^ CCR7^+^), effector memory T-cells (T_EM_; CD45RA^−^ CCR7^−^), effector memory RA T-cells (T_EMRA_; CD45RA^+^ CCR7^−^) and central memory T-cells (T_CM_; CD45RA^−^ CCR7^+^). In both young and older adults, the majority of pre-existing memory IFN-γ^+^ CD8^+^ T-cells prior to vaccination were either of the T_CM_ or T_EM_ phenotype (Supplementary Fig. 6). In young adults, both first and second vaccinations increased the frequency of antigen-specific IFN-γ^+^ CD8^+^ T_CM_ and T_EM_ CD8^+^ T-cells for up to 18 months after the first vaccination. In older adults, the increase in antigen-specific IFN-γ^+^ CD8^+^ T-cells after vaccination was observed only in the T_CM_ pool.

## 4 Discussion

We have developed viral vectored influenza vaccines, MVA-NP + M1 and ChAdOx1 NP + M1, to provide broad spectrum protection against influenza A virus by the boosting of pre-existing T-cell responses to conserved influenza antigens. In this report we demonstrate that a two-dose heterologous combination of these two viral vectored influenza vaccines is safe in young and older adults, significantly increases frequencies of cross-reactive T-cells and durably maintains these T-cells at high frequencies for 18 months after vaccination.

We had previously demonstrated the safety of single dose vaccinations with MVA-NP + M1 and ChAdOx1 NP + M1 vaccines in young and older adults, identified an optimal dose balancing reactogenicity and immunogenicity, and demonstrated the induction of influenza-specific T-cells following vaccination (Antrobus et al., 2014a; Antrobus et al., 2014b; Antrobus et al., 2012; Berthoud et al., 2011; Lillie et al., 2012). This study evaluated the combination of these two vaccines to identify the optimal order and interval in the vaccination schedule. We tested MVA-NP + M1 followed by ChAdOx1 NP + M1 (MVA/ChAdOx1) and ChAdOx1 NP + M1 followed by MVA-NP + M1 (ChAdOx1/MVA) with an interval of either 8 weeks or 52 weeks between the two vaccines. Both vaccination combinations were well-tolerated in young and older adults with mild to moderate injection site pain the most commonly reported adverse event. In young adults with pre-existing influenza-specific T-cell responses, all four vaccination regimes increased and maintained T-cell responses above pre-vaccination frequencies for 18 months. However, the MVA/ChAdOx1 regimen with either an 8 week or 52 week interval maintained T-cell responses at higher levels compared to the ChAdOx1/MVA regimen.

The long-term maintenance of T-cell responses is a critical component of successful vaccination strategies as T-cell responses are thought to be short lived following natural infection. A prospective study of T-cell responses after a symptomatic infection found rapid decline in responses within a few months after infection (Hillaire et al., 2011) while others have suggested that responses have a half-life of 2–3 years (McMichael et al., 1983a). More recent work has reported detection of T-cell responses many years after an infection, although it is unclear whether this was due to natural boosting in the intervening period.(van de Sandt et al., 2015) More importantly, detectable T-cell responses following natural infection are not necessarily present at protective levels. Although memory responses can still be boosted following natural infection, cohort and challenge studies indicate that there is a correlation between higher T-cell responses and reduction in symptom severity, viral shedding and illness duration (Hayward et al., 2015; McMichael et al., 1983b; Sridhar et al., 2013). This study shows that after vaccination with viral vectors, T-cell responses are maintained at high levels for a long period of time, raising the possibility that T cell mediated protection after vaccination could last for considerably longer than T-cell mediated protection after natural infection. This report is the longest follow-up of T-cell responses following influenza vaccination. Our study demonstrates long-term maintenance (up to 18 months) of T-cell responses with a heterologous viral vector vaccine strategy. Moreover, even a single dose of either viral vector vaccine in young adults can maintain T-cells above pre-vaccination frequencies for up to 1 year post-vaccination.

This long-term maintenance is critical in older adults in whom reducing severity of illness is paramount, particularly as seasonal influenza vaccines have reduced efficacy (McElhaney et al., 2016). In contrast to young adults, a single dose of ChAdOx1 did not durably maintain T-cell responses above pre-vaccination levels in older adults. However, the two-dose heterologous combination schedule of ChAdOx1/MVA boosted pre-existing T-cells and maintained these T-cells at significantly higher frequencies compared to pre-vaccination levels up to 8 months after the first vaccination. Longer follow-up of these responses is warranted. It is notable that reactogenicity after the ChAdOx1/MVA vaccination regimen was significantly lower in older adults compared to younger adults.

Our study showed no significant difference in generation and maintenance of immune responses between vaccination schedules with an 8 week or 52 week interval between doses. The two dose heterologous regimen administered 1 year apart is of particular interest to integrate into existing vaccination schedules and our data paves a development pathway for viral vectored vaccines administered in conjunction with existing strain-specific annual influenza vaccination. However, in older adults a shorter interval is likely to be needed as T-cell responses decline to pre-vaccination levels within 8 weeks after a single dose of ChAdOx1. In an outbreak scenario or pandemic setting, rapid induction of protective immune responses following vaccination is valuable to limit the severity and spread of the outbreak.

Studies conducted during the evolution of the H1N1 pandemic in 2009 have demonstrated that cross-reactive T-cells can provide protection against symptomatic pandemic influenza in the absence of cross-protective antibodies. We show that both ChAdOx1 NP + M1 and MVA-NP + M1 rapidly boost pre-existing cross-reactive T-cells to significantly higher levels within 1–3 weeks after vaccination. These responses remain above pre-existing frequencies up to 1 year post-vaccination, although MVA-NP + M1 induces significantly higher responses than ChAdOx1 NP + M1. A single dose of MVA-NP + M1, potentially stockpiled, is an attractive option for rapid development of T-cell immunity in the event of a pandemic to mitigate the severity of the pandemic, particularly in high-risk populations.

One limitation of this study is the lack of data on protective efficacy. However, in a previous experimental challenge study demonstrating reduced viral shedding and symptoms with a single dose of MVA-NP + M1, median IFN-γ ELISpot values of protected volunteers was 627 SFU/10^6^ PBMC. In comparison, long-term IFN-γ ELISpot responses at 18 months and peak responses were higher than this value (at least 1.25-fold greater) for all two dose vaccination regimes. Based on these data, we would speculate that a two-dose regimen is likely to confer higher levels of protection in a challenge study or efficacy trial. Further work is necessary to determine efficacy of these vaccines and the durability of protection. A key question in the potential deployment of our broadly protective T-cell vaccines is their use with existing inactivated and live vaccines, particularly in the elderly. Our study does not address this issue although further work is underway to assess strategies combining these viral vector vaccines with existing influenza vaccines (NCT03300362).

In conclusion, we have demonstrated that vaccines which stimulate T-cell responses against conserved antigens generate robust and durable immune responses that could confer broad and potentially long-lasting protection against influenza virus. This has the potential to profoundly impact both seasonal and pandemic influenza vaccination strategies, especially in high risk groups such as older adults.

## Role of the Funding Source

The funder had no role in study design, data collection, data analysis, data interpretation, or writing of the report. The corresponding and senior authors had access to all the data in the study and had final responsibility for the decision to submit for publication.

## Declaration of Interests

SG and AVH are co-founders of Vaccitech, a company developing viral vectored vaccines including broadly cross-reactive influenza vaccines. SS holds stock in Sanofi Pasteur which develops and markets influenza vaccines. HdG received a travel grant from Abbvie.

## Contributors

SG, AVH, conceived the study, RP, NV and SG wrote the protocol, and obtained all approvals. RP, NV, SS, PS and HdG implemented study procedures. LC, ME and BL conducted the laboratory work. LC and CQ conducted the primary statistical analysis (comparing the AUC of immune response between randomized arms groups 1 + 2 vs 3 + 4 and groups 5 vs 6), the secondary analyses (comparing peak response after vaccine 1 or 2 between pre-specified groups) and the post-hoc analyses (comparing all immune response in groups 1 + 2 vs 3 + 4, and the AUC in groups 1 + 3 and 2 + 4). AVH, DL and SNF acted as clinical principal investigators. All authors contributed to the interpretation of the data, writing of the report, and approved the final manuscript.

Table 1. Trial study design and participant demographics. G1 and G2 were first vaccinated with ChAdOx1 NP + M1 followed by a second MVA-NP + M1 vaccination 8 weeks later (W8; G1) or 52 weeks later (W52; G2). Volunteers in G3 and G4 received their first vaccination with MVA-NP + M1 followed by a second vaccination with ChAdOx1 NP + M1 at W8 or W52. G5 received ChAdOx1 NP + M1 alone and G6 received ChAdOx1 NP + M1 followed by an additional vaccination with MVA-NP + M1 at W8.

Table 2. Vaccine safety and reactogenicity. The number of volunteers experiencing local and systemic AEs after each vaccination. Only AEs with possible, probable or definite causal relationships are shown. The most common local AE was mild injection site pain and the most common systemic AE was mild fatigue and headache.

## Appendix A Supplementary Data

Supplementary material 1 Image 1

Supplementary material 2 Image 2
