# Corrigendum to “Heterologous Two-dose Vaccination with Simian Adenovirus and Poxvirus Vectors Elicits Long-lasting Cellular Immunity to Influenza Virus A in Healthy Adults” [EBioMedicine 29 (2018) 146–154]

**DOI:** 10.1016/j.ebiom.2018.05.001

**Published:** 2018-05-05

**Authors:** L. Coughlan, S. Sridhar, R. Payne, M. Edmans, A. Milicic, N. Venkatraman, B. Lugonja, L. Clifton, C. Qi, P.M. Folegatti, A.M. Lawrie, R. Roberts, H. de Graaf, P. Sukhtankar, S.N. Faust, D.J.M. Lewis, T. Lambe, A.V.S. Hill, S.C. Gilbert

**Affiliations:** a Icahn School of Medicine at Mount Sinai, Department of Microbiology, Annenberg Building, Room 16.30, One Gustave Levy Place, New York 10029, United States; b Centre for Statistics in Medicine, NDORMS, University of Oxford, Botnar Research Centre, Windmill Road, Oxford OX3 7LD, UK; c NIHR Wellcome Trust Clinical Research Facility, University of Southampton, University Hospital Southampton NHS Foundation Trust, Southampton, UK; d Clinical Research Centre, University of Surrey, Guildford GU2 7AX, UK; e The Jenner Institute, University of Oxford, ORCRB, Roosevelt Drive, Oxford OX3 7DQ, UK; f Sanofi Pasteur, MARCY l'ETOILE 69280, France

The authors wish to point out that L. Coughlan and S. Sridhar were both at the Jenner Institute, University of Oxford, OX3 7DQ, UK, when the work for the paper was completed.

