# Supplementary material for: Heterologous Two-Dose Vaccination with Simian Adenovirus and Poxvirus Vectors Elicits Long-Lasting Cellular Immunity to Influenza Virus A in Healthy Adults
Source: eBioMedicine. 2018 Feb 15;29:146–54. doi: 10.1016/j.ebiom.2018.02.011 (PMC5926543; doi:10.1016/j.ebiom.2018.02.011)
Supplement: Supplementary file 1 — Supplementary material 1 [file mmc1.pdf]

CLINICAL TRIAL PROTOCOL

Study reference: FLU005

A phase I study to determine the safety and immunogenicity of vaccination regimens employing the candidate influenza vaccines MVA-NP+M1 and ChAdOx1 NP+M1

Protocol Version Number:	3.3
Date:	30th July 2015
EudraCT Number:	2012-004626-25
Sponsor:	University of Oxford
Chief Investigator:	Prof Adrian Hill
Local Safety Monitor:	Dr Brian Angus

Investigator Agreement

"I have read this protocol and agree to abide by all provisions set forth therein. I agree to comply with the International Conference on Harmonisation Tripartite Guideline on Good Clinical Practice."

Chief Investigator

Date 26/8/2015

Professor Adrian Hill

Principal Investigator

8/2/2016

Date

S. [illegible]

Print name

Confidentiality Statement

This document contains confidential information that must not be disclosed to anyone other than the Sponsor, the Investigator Team, and members of the Independent Ethics Committee. This information cannot be used for any purpose other than the evaluation or conduct of the clinical investigation without the prior written consent of Prof Adrian Hill.

Study Code: FLU005

EudraCT Number: 2012-004626-25

NRES Committee South Central - Oxford A Reference Number:

Chief Investigator	Professor Adrian V S Hill<br>Centre for Clinical Vaccinology and Tropical Medicine<br>Churchill Hospital, Old Road, Headington<br>Oxford, OX3 7LE<br>Email: adrian.hill@ndm.ox.ac.uk
Principal Investigator	Dr Saul N Faust<br>Wellcome Trust Clinical Research Facility<br>University of Southampton<br>C Level, West Wing, Mailpoint 218<br>University Hospital Southampton NHS Foundation Trust, Tremona Road, Southampton, SO16 6YD<br>Email: s.faust@soton.ac.uk<br><br>Professor David Lewis<br>Surrey Clinical Research Centre<br>University of Surrey<br>Guildford<br>GU2 7XP<br>Email: d.j.lewis@surrey.ac.uk
Trial sites	Centre for Clinical Vaccinology and Tropical Medicine<br>Churchill Hospital, Old Road, Headington<br>Oxford, OX3 7LE<br>Tel: 01865 857417<br>Fax: 01865 857471<br><br>PI at Site: Professor Adrian Hill<br><br>NIHR Wellcome Trust Clinical Research Facility<br>University Hospital Southampton NHS Foundation Trust, Tremona Road, Southampton, SO16 6YD<br><br>PI at Site: Dr Saul Faust<br><br>Surrey Clinical Research Centre<br>University of Surrey<br>Guildford<br>GU2 7XP<br><br>PI at site: Professor David Lewis

Sponsoring Institution	University of Oxford<br>Clinical Trials & Research Governance<br>Joint Research Office, Block 60<br>Churchill Hospital, Old Road<br>Headington, Oxford, OX3 7LE<br>Tel: 01865 572224<br>Fax: 01865 572228<br>Email: ctrg@admin.ox.ac.uk
External Monitor	University of Oxford<br>Clinical Trials and Research Governance<br>Joint Research Office, Block 60<br>Churchill Hospital, Old Rd<br>Headington, Oxford, OX3 7LE<br>Tel: 01865 572224<br>Fax: 01865 572228<br>Email: ctrg@admin.ox.ac.uk
Local Safety Monitor	Dr Brian Angus<br>Centre for Clinical Vaccinology and Tropical Medicine<br>Churchill Hospital, Old Road, Headington<br>Oxford, OX3 7LE<br>Tel: 01865 553 20289<br>Email: brian.angus@ndm.ox.ac.uk

Revision History

Version	Date	Author(s)
1.0	06.12.2012	Ruth Payne, Richard Antrobus
1.1	26.02.2013	Ruth Payne
2.0	26.03.2013	Ruth Payne
2.1	18.04.2013	Ruth Payne
2.2	01.05.2013	Ruth Payne
2.3	11.06.2013	Ruth Payne
3.0	09.05.2014	Sarah Gilbert, Navin Venkatraman
3.1	17.06.2014	Navin Venkatraman
3.2	28.01.2015	Saranya Sridhar
3.3	30.07.2015	Emma Bakpa

Details of Revisions Made

Version	Section	Modifications
1.1	8.5	Wording changed to reflect MHRA guidelines- Sponsor to be informed within 24 hours of an SAE occurring. Details of procedures for informing the Sponsor and Local Safety Monitor clarified.
2.0	4.1, 5.1, 6.1, 7.3, 7.6, 8.5 and 13.2	Trial site added (Southampton). Changes made regarding this to abbreviations, synopsis (plus correction to randomisation section), Sections 4.1, 5.1 (plus corrections to concentration of ChAdOx1 NP+M1 and adding batch/ lot numbers for both vaccines), 6.1, 7.3, 7.6 and 8.5.<br><br>Section 13.2- Chief Investigator added to list of people who will have access to records.<br><br>Grammatical errors corrected throughout the document.<br><br>Safety bloods added at day 0

2.1	7.7	Table 3- typographical error corrected- AEs reviewed throughout study
2.2		PI investigator signature section added (Page 2)
2.3	5.1	SOP name updated from OVCSC008 to VM006
3.0	2.3<br>4.1, 4.2, 4.4, 4.6<br>6.1<br>6.3<br>7.3<br>7.4, 7.5<br>7.7<br>8.3<br>8.5<br>9	Update of previous experience with ChAdOx1 NP+M1<br>Principal Investigator and trial site added (Surrey) - Changes made regarding this to synopsis in addition to the sections detailing Study Overview. Groups 5 and 6 added<br>Surrey Clinical Research Database added to methods of recruitment<br>New inclusion and exclusion criteria added for Groups 5&6<br>Addition of Surrey Pathology Services (SPS) and St Helier hospital for sample analysis<br>Minor changes regarding pregnancy testing and contraception<br>Schedule of visits and tables added for Groups 5&6<br>Table for grading of AEs deleted; replaced with site-specific tables stored in TMF<br>SOP name updated for reporting of serious adverse events<br>Addition of statistical analysis for Groups 5&6
3.1	Tables 5 & 6	Blood volume for all haematology samples reduced from 2.5ml to 2ml for all Surrey volunteers
3.2	Tables 3 and 4	Table 3. Typographical error corrected – Window period +21 for W52 visit changed to ± 21<br>Table 4 Typographical error corrected – Window period + 21 for W8 visit changed to ± 21
3.3	13.1	Change to details regarding OpenClinica data storage

Abbreviations

**AE**	Adverse event
**AR**	Adverse drug reaction
**CBF**	Clinical Biomanufacturing Facility
**CCVTM**	Centre for Clinical Vaccinology and Tropical Medicine
**CEF**	Chick Embryo Fibroblast cells
**ChAd63**	Chimpanzee Adenovirus 63
**ChAdOx1**	Chimpanzee Adenovirus Ox1
**CRF**	Case Report Form
**ELISA**	Enzyme-linked immunosorbant assay
**ELISpot**	Enzyme-linked immunospot
**FBC**	Full blood count
**GCP**	Good Clinical Practice
**GMO**	Genetically modified organism
**HA/H**	Haemagglutinin
**HBsAg**	Hepatitis B Surface Antigen
**HCG**	Human Chorionic Gonadotrophin
**HCV**	Hepatitis C virus
**HIV**	Human immunodeficiency virus
**HLA**	Human leukocyte antigen
**ICH**	International Conference on Harmonisation
**IB**	Investigators Brochure
**ID**	Intradermal
**IM**	Intramuscular
**LSM**	Local safety monitor
**M1**	Matrix protein 1
**MHRA**	Medicines and Healthcare products Regulatory Agency
**MVA**	Modified Vaccinia virus Ankara
**MVA NP+M1**	Recombinant Modified Vaccinia virus Ankara expressing influenza nucleoprotein fused to matrix protein 1
**NA/N**	Neuraminidase
**NHS**	National Health Service
**NIHR**	National Institute for Health Research
**NP**	Nucleoprotein
**pfu**	Plaque forming units
**REC**	Regional Ethics Committee
**RNA**	Ribonucleic acid
**SAE**	Serious adverse event
**SOP**	Standard Operating Procedure
**SUSAR**	Suspected unexpected serious adverse reaction
**TIV**	Trivalent inactivated influenza vaccine
**TRAP**	Thrombospondin related adhesion protein
**vp**	Viral particles
**WTCRF**	Wellcome Trust Clinical Research Facility, Southampton

Contents

1. Synopsis 11
2. Background and Rationale 12
2.1 The need for a new vaccine against influenza 12
2.2 The selection of NP+M1 as an insert for a viral vectored vaccine 13
2.3 Previous experience with viral vectored vaccines expressing NP+M1 13
ChAdOx1 NP+M1 (see also ChAdOx1 IB) 13
MVA-NP+M1 (see also MVA-NP+M1 IB) 16
3. Objectives 18
4. Study Design 19
4.1 Study Overview 19
4.2 Randomisation 20
4.3 Study endpoints 20
4.4 Duration of study 20
4.5 Definition of the Start and End of the Trial 20
4.6 Potential Risks 20
Local reactions 20
Systemic reactions 20
4.7 Known Potential Benefits 21
5. Study Vaccines 22
5.1 Vaccine formulation, storage and accountability 22
ChAdOx1 NP+M1 22
MVA NP+M1 22
5.2 Vaccine administration 22
5.3 Minimising environmental contamination with Genetically Modified Organisms (GMO) 22
6. Recruitment and Withdrawal of Volunteers 23
6.1 Volunteers 23
6.2 Informed Consent 23
6.3 Inclusion and Exclusion Criteria 24
Inclusion Criteria for Groups 1-4 24
Exclusion Criteria for Groups 1-4 24
Inclusion Criteria for Groups 5-6 25
Exclusion Criteria for Groups 5-6 25
6.4 Prevention of over-volunteering 26
6.5 Postponement Criteria 26
6.6 Re-vaccination exclusion criteria 26

6.7 Withdrawal Criteria 27
7. Treatment of Trial Volunteers 28
7.1 Study Procedures 28
7.2 Observations 28
7.3 Blood Tests 28
7.4 Urinalysis 28
7.5 Contraception 28
7.6 Vaccinations 29
7.7 Study visits 29
Screening visit 29
Day 0- Enrolment and First Vaccination 29
Groups 1-4 29
Safety Review – Day 1 29
Follow up visits- weeks 1, 2, 3, 4, 8, (9, 10, 11) 26, 52, (53, 54, 55) 78 30
Groups 5 and 6 30
Safety Review – Day 2 30
Follow up visits- Days 7, 14, 28, 56, (63, 84) 182 30
8. Assessment of Safety 35
8.1 Definitions 35
Adverse Event (AE) 35
Adverse Reaction (AR) 35
Unexpected Adverse Reaction 35
Serious Adverse Event (SAE) 35
Serious Adverse Reaction (SAR) 36
Suspected Unexpected Serious Adverse Reaction (SUSAR) 36
Foreseeable Adverse Reactions 36
8.2 Causality Assessment 37
8.3 Severity Assessment 38
8.4 Procedures to be followed in the event of abnormal findings 39
8.5 Reporting Procedures for all Adverse Events 39
Reporting Procedures for Serious Adverse Events 39
Reporting Procedures for SUSARs 39
8.6 Local Safety Monitor 40
8.7 Pregnancy 40
9. Statistics 41
Power Calculations 41

10. Serious Breaches ... 42
11. Quality Control and Quality Assurance Procedures ... 42
12. Ethics ... 43
12.1 Good Clinical Practice (GCP) ... 43
12.2 Ethical Review ... 43
12.3 Informed Consent ... 43
13. Data Handling and Record Keeping ... 44
13.1 Data Handling ... 44
13.2 Record Keeping ... 44
13.3 Source Data and Case Report Forms (CRFs) ... 44
13.4 Data Protection ... 44
14. Financing and Insurance ... 45
14.1 Financing ... 45
14.2 Insurance ... 45
14.3 Compensation ... 45
15. References ... 46

1. Synopsis

**Trial Title**	A phase I study to determine the safety and immunogenicity of vaccination regimens employing the candidate influenza vaccines MVA-NP+M1 and ChAdOx1 NP+M1
**Trial Centres**	Centre for Clinical Vaccinology and Tropical Medicine (CCVTM)Old Road, Headington, Oxford, OX3 7LE, UK<br><br>Wellcome Trust Clinical Research Facility, Southampton University Hospitals NHS Trust, Southampton, SO16 6YD<br><br>Surrey Clinical Research Centre, University of Surrey, Guildford, GU2 7XP
**Trial Identifier**	FLU005
**Clinical Phase**	I
**Trial Design**	Phase I randomised observational study
**Trial Population**	Healthy adults aged 18-50 in Groups 1-4<br><br>Healthy adults aged 50 or above in Groups 5 and 6
**Randomisation**	Randomised in variable block sizes according to vaccine allocation but not according to interval duration
**Planned Sample Size**	72 volunteers, in 6 groups of 12
**Follow-up duration**	Approximately 18 months for Groups 1-4<br><br>Approximately 6 months for Groups 5 and 6
**Planned Trial Period**	Approximately 2.5 years
**Primary Objective**	To assess the safety of ChAdOx1-NP+M1 in a prime-boost regime with MVA NP+M1 and compare immunogenicity of the vaccines
**Investigational Product**	ChAdOx1-NP+M1<br><br>MVA NP+M1
**Form**	Liquid
**Doses**	ChAdOx1 NP+M1: 2.5 x 10^{10} vp<br><br>MVA NP+M1: 1.5 x 10^{8} pfu
**Route**	Intramuscular (IM) injection into the deltoid

2. Background and Rationale

2.1 The need for a new vaccine against influenza

Influenza is an orthomyxovirus and encodes a segmented RNA genome. Influenza is divided into 3 groups- A, B and C. Most seasonal influenza and all known pandemics are caused by Influenza A, whilst B and C cause low-levels disease and sporadic outbreaks [1]. Influenza A is subdivided further on the basis of haemagglutinin (H or HA) and neuraminidase (N or NA) activity- for example, H1N1 subtype. There are at least 16 different types of HA and 9 of NA. These proteins are found as spiked surface projections on the Influenza A virus [2]. Other genes encode proteins vital for structure, reproduction and virulence including nucleoprotein, M1 (matrix), M2 (ion pore), NS1, NS2, PA, PB1, PB1-F2 and PB2, which are found within the envelope [1, 2].

Seasonal influenza has a huge annual impact worldwide, accounting for tens of millions of illnesses, hundreds of thousands of excess hospitalizations, and tens of thousands of excess deaths in the US alone [3]. In addition, influenza can also occur in pandemics and the infection of humans by avian influenza (H5N1) could trigger a new pandemic if the virus acquires the ability to transmit from person to person [4]. Current influenza vaccines work by inducing antibodies against the highly polymorphic surface proteins (haemagglutinin, neuraminidase) of the influenza virus. The vaccine consists of proteins from three influenza viruses (H1N1, H3N2 and influenza B) reformulated annually to keep up with genetic drift in the surface proteins of these seasonal viruses. As these proteins are highly polymorphic, there is very little or no cross-subtype (or heterosubtypic) protection and limited cross-strain protection even within subtypes. Approximately one year in 20, vaccine efficacy is much lower than expected owing to antigenic drift away from the vaccine strain [5]. Efficacy is substantially reduced in older adults, who form one of the main target groups for vaccination: vaccination prevents laboratory-confirmed influenza in 70–90% of young adults, but in only 30–40% of older adults [6]. This need for constant redesign and remanufacture increases the cost of the vaccines, places limitations on supply [7], and most importantly means that vaccines for newly arising strains can only be produced once the HA and NA sequences of viruses posing the greatest threat to human health have been identified.

A vaccine against influenza that induced protective T cell responses against conserved internal antigens could provide lasting immunity against not only human seasonal influenza, but also other subtypes currently found in avian species or swine which have the potential to cause a new pandemic. Recombinant viral vectored vaccines can be used in humans to generate strong CD4 and CD8 T cell responses to a wide range of antigens. Since adults have been primed by prior exposure to influenza, a viral vector expressing conserved internal antigens of influenza such as NP and matrix protein 1 (M1) could be used to boost cross-reactive T-cell responses to protective levels, providing broad immunity to all subtypes of influenza A [8].

Adenoviruses are the ideal vector for such a vaccine as they induce broad, potent and well-maintained T cell responses after a single vaccination so could be used to confer broad immunity rapidly. Furthermore they possess a genetically stable virion (so that inserts of foreign genes are not deleted); they can infect large numbers of cells and the transferred information remains epichromosomal, thus avoiding any potential for insertional mutagenesis. Replication defective adenovirus can be engineered by deletion of genes from the E1 locus, which is required for viral replication, and these viruses can be propagated easily with good yields in cell lines expressing E1 from AdHu5 such as human embryonic kidney cells 293 (HEK 293) [9]. Previous mass vaccination campaigns using orally administered live human adenovirus serotype 4 and 7 in large numbers of US military personnel have shown good safety and efficacy data [10].

Human adenoviruses have been used as vaccine vectors for a number of conditions; however a limiting factor to widespread use has been the level of anti-vector immunity present in humans where adenovirus is a

ubiquitous infection. This has led to the consideration of simian adenoviruses, which are not known to cause pathology or illness in humans and to which the prevalence of antibodies is low. Several studies have been carried out previously in Oxford using the simian viral vector chimpanzee adenovirus 63 (ChAd63), expressing various malaria antigens, which have shown excellent immunogenicity and safety. The new vector ChAdOx1 has been developed within the Jenner Institute from another chimpanzee adenovirus. Seroprevalence of antibodies to ChAd63 and ChAdOx1 has been examined in both the UK and Gambian populations, with the result that no individuals with high titre neutralising antibodies were found in the UK group, and very low numbers in the Gambian group, which compares very favourably to Ch63 tested at the same time ([11], and supplementary data Fig. 1). By using a simian adenovirus such as ChAdOx1, reduction in immunogenicity of the vaccine caused by prior natural exposure to the vector is avoided, and efficient induction of CD8+ T cells, which are the most relevant T cell lymphocyte population for the clearance of intracellular pathogens can be achieved by a single immunisation.

Vaccination with MVA-NP+M1 results in a rapid increase in influenza-specific effector T cells, which declines equally rapidly to an intermediate level, with a continued slow decline over the course of a year [8]. In contrast, following simian adenovirus vectored malaria vaccine administration, after the initial rapid peak and decline, there is no further decrease over the course of a year [12]. The hypothesis in this study is therefore that following vaccination with ChAdOx1 NP+M1 the T cell response will be better maintained, resulting in a longer period of protection. Furthermore, the response induced by vaccination with either vector may then be boosted by a second vaccination with the other vector.

2.2 The selection of NP+M1 as an insert for a viral vectored vaccine

There is very little polymorphism of NP and M1 between influenza A isolates. NP is 92% identical between H3N2 and H1N1 strains, and 91% identical between H3N2 and H5N1 strains. M1 is 95% identical between H3N2 and H1N1 strains, and 93% identical between H3N2 and H5N1 strains. This low level of variation appears to allow strong T cell cross-reactivity. For comparison, the sequence of the TRAP antigen used in similar malaria vaccine candidates differs by 8% from the sequence in the challenge strain, and excellent immunological cross-reactivity and cross-strain protection has been observed [11].

2.3 Previous experience with viral vectored vaccines expressing NP+M1

ChAdOx1 NP+M1 (see also ChAdOx1 IB)

ChAdOx1 is a novel recombinant chimpanzee adenovirus designed as a vaccine vector. It has recently been used in a Phase 1 dose escalation study in Oxford (FLU004), in which it was administered to 15 volunteers. The dose was escalated from 5 x 10^8 vp to 5 x 10^{10} vp with safety reviews at each dose escalation (5 x 10^8 vp, 5 x 10^9 vp, 2.5 x 10^{10} vp and 5 x 10^{10} vp).The study demonstrated that ChAdOx1-NP+M1 at a dose of 5 x 10^{10} vp is safe in healthy volunteers but had an unacceptable reactogenicity profile with 2 out of 6 volunteers who received this dose experiencing severe local and systemic reactions. The decision has therefore been made to use a dose of 2.5 x 10^{10} vp in this study, to balance immunogenicity and reactogenicity. There were no serious adverse events during the trial, and no severe reactions with the 2.5 x 10^{10} vp dose. Most adverse events reported by volunteers were mild or moderate in nature throughout the whole trial. Figures 1 and 2 show the local and systemic reactions reported by the 3 volunteers in FLU004 who received ChAdOx1-NP+M1 at a dose of 2.5 x 10^{10} vp. Immunogenicity in these 3 volunteers, as measured by ELISpot response, is shown in Figures 3 and 4. ChAdOx1-NP+M1 is immunogenic, with an increase in median ELISpot response at Day 14 (relative to vaccination at Day 0), sustained to Day 21.

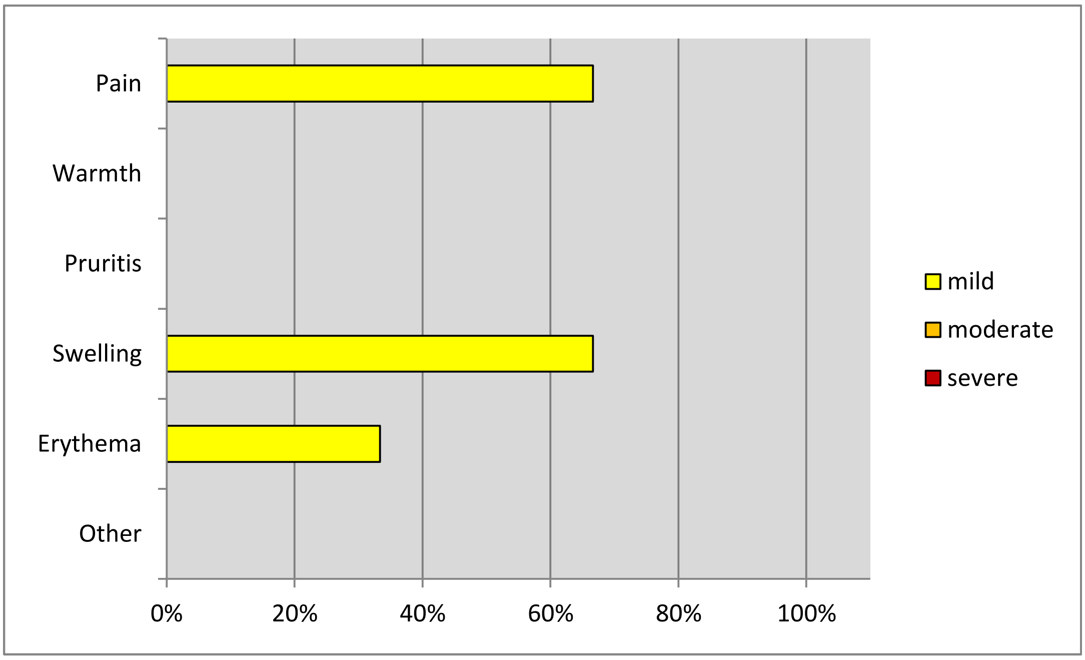

Figure 1: *Local adverse events with ChAdox1-NP+M1 2.5 x 10^{10} vp*

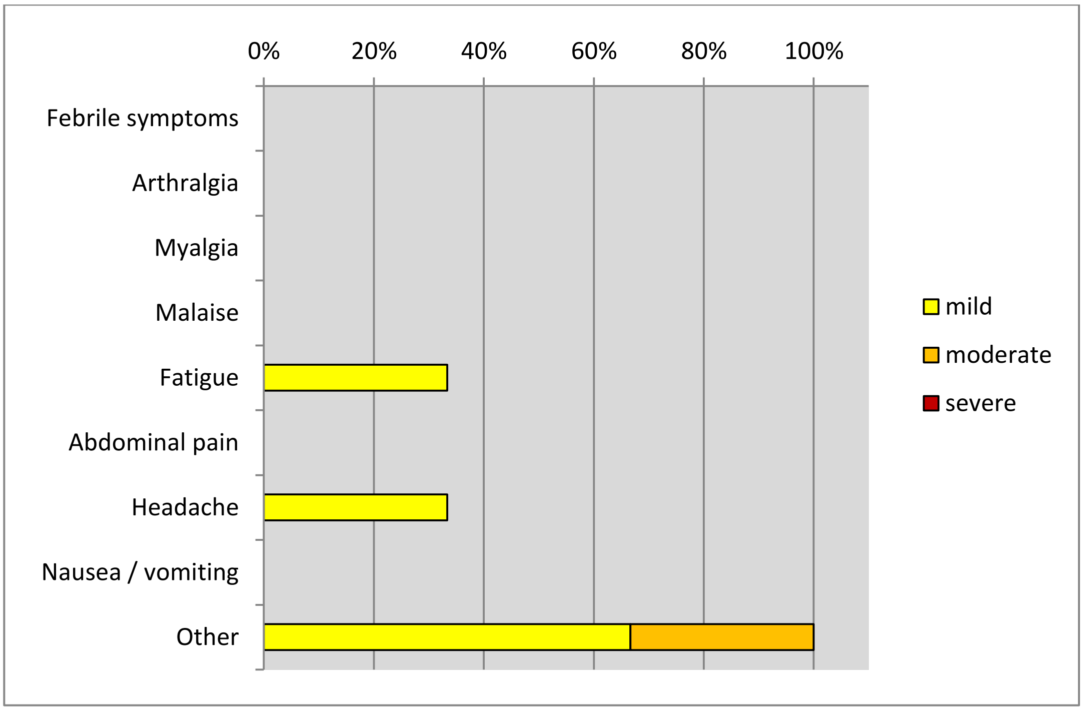

Figure 2: *Systemic adverse events with ChAdOx1-NP+M1 2.5 x 10^{10} vp*

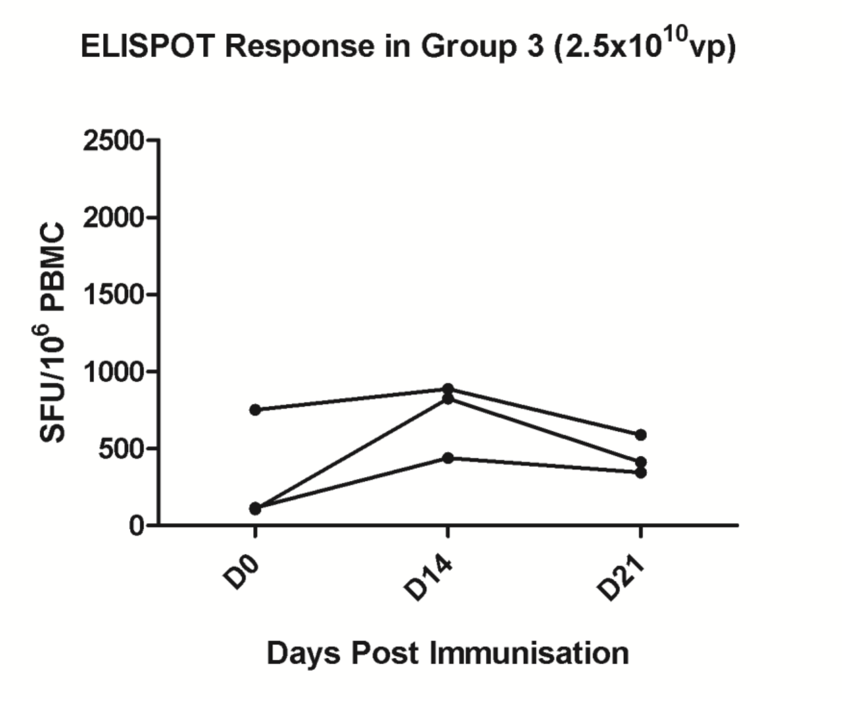

Figure 3: *ELISpot response in volunteers given ChAdOx1-NP+M1 2.5 x 10^{10} vp*

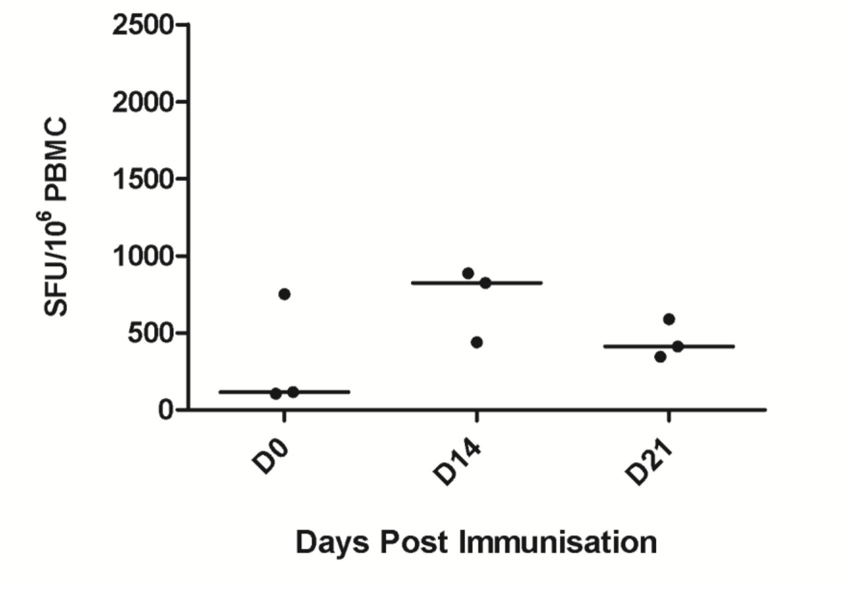

Figure 4: *Median ELISpot response to ChAdOx1-NP+M1 2.5 x 10^{10} vp*

MVA-NP+M1 (see also MVA-NP+M1 IB)

Modified Vaccinia virus Ankara (MVA) expressing NP and M1 (MVA NP+M1) has already been shown to provide protection against infection in an influenza Phase IIa challenge study (FLU002). It has also been given at the same time as the standard seasonal influenza vaccine (FLU003) and has been shown to have a good safety profile. The vaccine was safe and boosted T cell responses as expected [8]. There have not been any vaccine related serious adverse events during these trials. A dose dependent increase in adverse events was observed and a dose of 1.5 $x10^8$ pfu was found to be the optimal balance between immunogenicity and reactogenicity [8]. In the Phase IIa influenza challenge study, fewer vaccinated volunteers developed influenza than the unvaccinated volunteers and there was a statistically significant reduction in duration of virus shedding in vaccinated volunteers [13]. Thus the approach appears to have promise, but in other clinical studies of malaria vaccines conducted in parallel, simian adenovirus-vectored vaccines have been found to be a more potent system for inducing and boosting T cell responses to the recombinant antigens that they express [12].

MVA-NP+M1 has been administered to over 80 individuals across a range of doses and via both the intramuscular and intradermal routes, as shown in Table 1. Figure 5 demonstrates the immunogenicity (as determined by interferon-gamma ELISpot) of MVA-NP+M1 in older adults (aged 50+) receiving a dose of 1.5 $x10^8$ pfu.

Country	Study	Vaccine	Age	Route	Dose	Number of volunteers
UK	FLU001	MVA-NP+M1	18-50	ID	5 $x10^7$ pfu	12
		MVA-NP+M1	18-50	IM	5 $x10^7$ pfu	8
		MVA-NP+M1	18-50	IM	2.5 $x10^8$ pfu	8
		MVA-NP+M1	50-59	IM	1.5 $x10^8$ pfu	10
		MVA-NP+M1	60-69	IM	1.5 $x10^8$ pfu	10
		MVA-NP+M1	70+	IM	1.5 $x10^8$ pfu	10
UK	FLU002	MVA-NP+M1	18-50	IM	1.5 $x10^8$ pfu	15
UK	FLU003	MVA-NP+M1 (together with seasonal influenza vaccine)	50+	IM	1.5 $x10^8$ pfu	9
UK	FLU004	ChAdOx1-NP+M1	18-50	IM	5 x 10^8 vp	3
					5 x 10^9 vp	3
					2.5 x 10^{10} vp	3
					5 x 10^{10} vp	6

Table 1: *Studies to date using viral vectored vaccines expressing NP+M1*

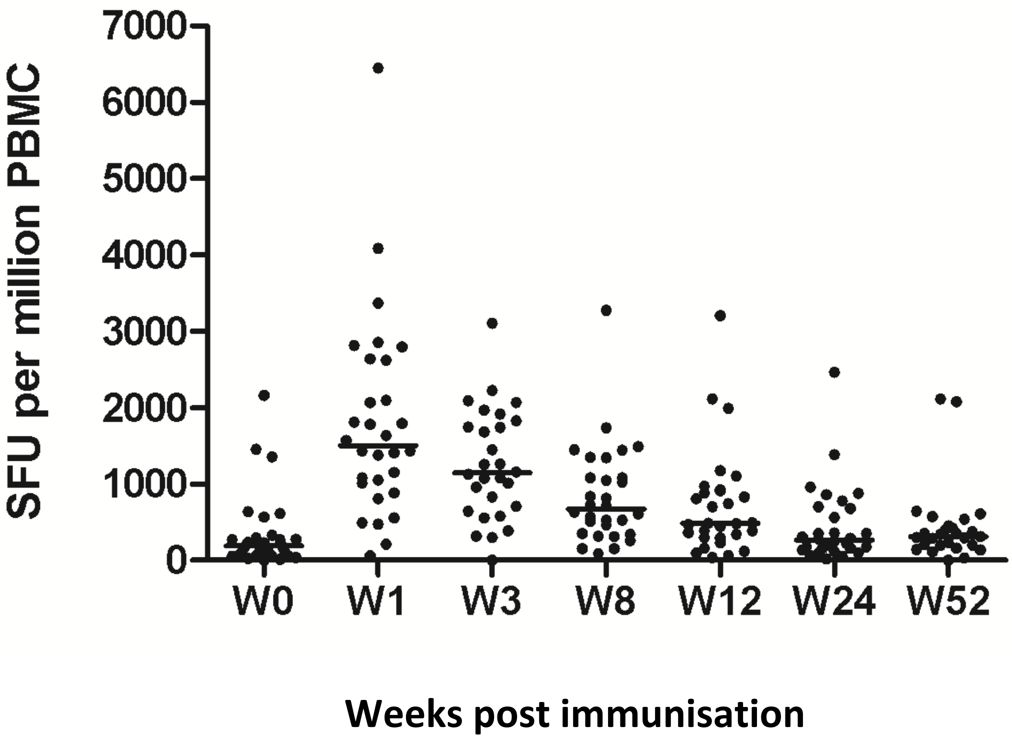

Figure 5: *Immunogenicity of MVA-NP+M1 in older adults*

3. Objectives

1. To assess the safety of prime/boost vaccination regimens employing MVA-NP+M1 and ChAdOx1 NP+M1.
2. To assess the cellular immune response generated by prime/boost vaccination regimens employing MVA-NP+M1 and ChAdOx1 NP+M1.

4. Study Design

4.1 Study Overview

This will be a randomised observational phase I study in 48 healthy volunteers aged 18-50 and 24 healthy volunteers aged 50 or over. The study is assessing safety and immunogenicity of viral vectored vaccines ChAdOx1 NP+M1 and MVA NP+M1 in heterologous prime-boost regimens. A crossover design will allow comparison of the two vaccines. Volunteers will be divided into 6 groups (n=12 in each group). Groups 1-4 will be recruited simultaneously as will Groups 5 and 6 to control for seasonal changes in influenza. This is because at certain times of year there is likely to be a higher naturally acquired T cell response to influenza than at other times due to circulating influenza virus in the community.

The vaccines have both been used safely in humans before. A recent dose escalation study has demonstrated that a ChAdOx1 NP+M1 dose of 2.5 x 10^{10}vp is safe and immunogenic. Previous studies in Oxford have demonstrated that the optimal dose of MVA NP+M1 is 1.5 x 10^{8}pfu, balancing immunogenicity and reactogenicity. Both vaccines are administered intramuscularly.

48 volunteers aged 18-50 (Group 1-4) will be recruited and randomised in variable block sizes to receive ChAdOx1 NP+M1 or MVA NP+M1 prime at day 0. 24 individuals will be boosted 8 weeks later, and the remaining 24 volunteers will be boosted 52 weeks later, with the other vaccine. Additionally, 24 volunteers aged 50 or over will be randomised to group 5 or 6, receiving ChAdOx 1 NP+M1 on day 0, with a boost of MVA-NP+M1 8 weeks later for group 6 only. Groups are set out in Table 2 with the boosting regimens.

	**Day 0**	**Week 8**	**Week 52**
**Group 1**	ChAdOx1 NP+M1	MVA NP+M1	--
**Group 2**	ChAdOx1 NP+M1	--	MVA NP+M1
**Group 3**	MVA NP+M1	ChAdOx1 NP+M1	--
**Group 4**	MVA NP+M1	--	ChAdOx1 NP+M1
**Group 5 (age 50 plus)**	ChAdOx1 NP+M1		
**Group 6 (age 50 plus)**	ChAdOx1 NP+M1	MVA NP+M1	

Table 2: *Study groups*

Vaccines will be administered at the Centre for Clinical Vaccinology and Tropical Medicine (CCVTM) in Oxford, the WTCRF, Southampton and the Surrey Clinical Research Centre, University of Surrey. Volunteers in groups 1 to 4 will be followed up to week 78 and volunteers in groups 5 and 6 will be followed up to week 26, with clinic visits and blood tests as outlined in section 7.

4.2 Randomisation

A statistician at the Centre for Statistics in Medicine, Oxford will do the randomisation using variable block sizes and prepare sealed envelopes containing the allocations.

For groups 1-4, volunteers will initially be randomised to one of two groups. They will receive ChAdOx1 NP+M1 or MVA NP+M1 at day 0. Volunteers will **not** be randomised to specific study groups (i.e. groups 1-4) in order to allow volunteers to choose the intervals at which they are vaccinated (i.e. either day 0 and week 8 or day 0 and week 52). Volunteers recruited once groups are full will obviously not be able to choose those groups.

For groups 5 and 6, volunteers will be randomised to these groups using variable block sizes with equal allocation to the two groups.

4.3 Study endpoints

The specific endpoints for safety and reactogenicity will be actively and passively collected data on adverse events.

Immunogenicity endpoints will include interferon-gamma ELISpot as a marker of cell-mediated immunity.

4.4 Duration of study

Volunteers in groups 1-4 will all be followed up to week 78, and volunteers in groups 5 and 6 will be followed up to week 26 with clinic visits and blood tests as outlined in section 7.7 (Tables 3-6).

4.5 Definition of the Start and End of the Trial

The start of the trial is defined as the date of the first vaccination of the first volunteer. The end of the trial is the date of the last visit of the last volunteer.

4.6 Potential Risks

The general risks to participants in this Phase I study are associated with phlebotomy and with vaccination. The maximum volume of blood drawn over the 78-week study period (approximately 789 mL) should not compromise otherwise healthy volunteers. Potential risks include local and systemic reactions, which are described below.

In general, recombinant adenoviral and MVA vectors are safe. Similar vaccines encoding different antigens have been given to several thousand volunteers (including children) with a good safety profile.

Local reactions

Vaccination usually precipitates a local inflammatory reaction. This may include redness, swelling, scaling, tenderness, or itching. In previous studies using recombinant adenoviral vectored vaccines, these local reactions have spontaneously resolved typically within 1-2 weeks. Mild tenderness, bruising, light-headedness or, rarely, syncope, may also occur as result of venepuncture.

Systemic reactions

Systemic reactions that could potentially occur following immunisation with a recombinant adenoviral vaccine include a flu-like illness with feverishness, fatigue, malaise, arthralgia, myalgia and headache. As with any

other vaccine, the Guillain-Barré syndrome, (GBS) or immune mediated reactions that can lead to organ damage may (rarely) occur. As with any vaccine, serious allergic reactions may occur.

4.7 Known Potential Benefits

Volunteers will not benefit directly from participation in this study. However, it is hoped that the information gained from this study will contribute to the development of a safe and effective influenza vaccine regimen. The only benefits for participants would be information about their general health status.

5. Study Vaccines

5.1 Vaccine formulation, storage and accountability

ChAdOx1 NP+M1

ChAdOx1-NP+M1 is manufactured under Good Manufacturing Practice conditions at the Clinical Biomanufacturing Facility (CBF), Oxford. The vaccine is supplied as a liquid in glass vials for intramuscular administration and is stored, between –70°C and –90°C, in a locked freezer, at the clinical sites. All movements of the study vaccines between CBF and clinical sites will be documented. Vaccine accountability, storage, shipment and handling will be in accordance with SOP VM006 and other relevant local SOPs and forms.

Each vial of ChAdOx1-NP+M1 (Batch Number 04D11-01) contains 650 microlitres volume at a concentration of 1.12 x10^{11} vp/ml. ChAdOx1-NP+M1 is formulated in 10mM Histidine, 7.5% sucrose, 35mM NaCl, 1mM MgCl2 , 0.1% PS80, 0.1mM EDTA and 0.5% ethanol with a pH of 6.6.

The dose of ChAdOx1-NP+M1 to be used in this study will be 2.5 x 10^{10}vp

MVA NP+M1

MVA-NP+M1 is manufactured under Good Manufacturing Practice conditions by IDT Biologika GmbH, Germany. The vaccine is supplied as a liquid in glass vials for intramuscular administration and is stored, between –70°C and –90°C, in a locked freezer, at the clinical sites. All movements of the study vaccines between IDT and the clinical sites, and between the locked freezer and clinic room will be documented. Vaccine accountability, storage, shipment and handling will be in accordance with SOP VM006 and other relevant local SOPs and forms.

Each vial of MVA-NP+M1 (Lot Number 010907) contains 700 microlitres volume at a concentration of 1.3 x10^{8} pfu/ml in 10mM Tris buffer.

The dose of MVA-NP+M1 to be used in this study will be 1.5 x10^{8} pfu.

5.2 Vaccine administration

On vaccination day, vaccines will be allowed to thaw to room temperature and administered within 1 hour. The vaccine will be administered intramuscularly into the deltoid muscle of the arm. The doctor/nurse will wear gloves and an apron. For administration of the second vaccine (at 8 weeks or 52 weeks) the vaccine will be given into the opposite arm.

All volunteers will be observed in the unit for 1 hour (±10 minutes) after vaccination. During administration of the vaccine, medicines and resuscitation equipment will be immediately available for the management of anaphylaxis. Temperature, blood pressure and pulse will be measured before and after vaccination.

5.3 Minimising environmental contamination with Genetically Modified Organisms (GMO)

In order to minimise dissemination of the recombinant virus into the environment, the inoculation site will be covered with a dressing after immunisation. This should absorb any virus that may leak out through the needle track. The dressing will be removed from the injection site after 30 minutes (± 10 minutes) and will be disposed of as GMO waste by autoclaving, in accordance with the relevant Standard Operating Procedure (SOP) and current standard UK practice.

6. Recruitment and Withdrawal of Volunteers

6.1 Volunteers

Volunteers may be recruited by use of an advertisement +/- registration form formally approved by the ethics committee and distributed or posted in the following places:

- In public places with the agreement of the owner / proprietor
- In newspapers or other literature for circulation
- On radio via announcements
- On a website or social media site operated by our group or with the agreement of the owner or operator (including on-line recruitment through our website)
- By e-mail distribution to a group or list only with the express agreement of the network administrator or with equivalent authorisation.
- By email distribution to individuals who have already expressed an interest in taking part in any clinical trial at the Oxford Vaccine Centre.
- On stalls or stands at exhibitions or fairs
- Via presentations (e.g. presentations at lectures or invited seminars)
- Direct mail-out: This will involve obtaining names and addresses of adults via the most recent Electoral Roll. The contact details of individuals who have indicated that they do not wish to receive postal mail-shots would be removed prior to the investigators being given this information. The company providing this service is registered under the Data Protection Act 1998. Investigators would not be given dates of birth or ages of individuals but the list supplied would only contain names of those aged between 18-50 years, or alternatively those over 50 years (as per the inclusion criteria).
- Oxford Vaccine Centre databases: We may contact individuals from databases of groups within the CCVTM, (including the Oxford Vaccine Centre database) of previous trial participants who have expressed an interest in receiving information about all future studies for which they may be eligible.
- WTCRF Database of Healthy Volunteers: We may contact individuals from this database who have previously expressed an interest in receiving information about future studies for which they may be eligible.
- Surrey Clinical Research Centre Volunteer Database: We may contact individuals from this database who have previously expressed an interest in receiving information about future studies for which they may be eligible.

6.2 Informed Consent

All volunteers will sign and date the informed consent form before any study specific procedures are performed. The information sheet will be made available to the volunteer at least 24 hours prior to the screening visit. At the screening visit, the volunteer will be fully informed of all aspects of the trial, the potential risks and their obligations. The following general principles will be emphasised:

- Participation in the study is entirely voluntary
- Refusal to participate involves no penalty or loss of medical benefits
- The volunteer may withdraw from the study at any time

- The volunteer is free to ask questions at any time to allow him or her to understand the purpose of the study and the procedures involved
- The study involves research of an investigational vaccine
- There is no direct benefit for participating
- The volunteer's GP will be contacted to corroborate their medical history

The aims of the study and all tests to be carried out will be explained. The volunteer will be given the opportunity to ask about details of the trial, and will then have time to consider whether or not to participate. If they do decide to participate, they will sign and date two copies of the consent form, one for them to take away and keep, and one to be stored in the Case Report Form (CRF). These forms will also be signed and dated by the one of the Investigators.

6.3 Inclusion and Exclusion Criteria

Inclusion Criteria for Groups 1-4

The volunteer must satisfy all the following criteria to be eligible for the study:

- Healthy adults aged 18 to 50 years
- Able and willing (in the Investigator's opinion) to comply with all study requirements
- Willing to allow the investigators to discuss the volunteer's medical history with their General Practitioner
- For females only, willingness to practice continuous effective contraception during the study and a negative pregnancy test on the day(s) of vaccination
- Agreement to refrain from blood donation during the course of the study
- Provide written informed consent

Exclusion Criteria for Groups 1-4

The volunteer may not enter the study if any of the following apply:

- Participation in another research study involving an investigational product in the 30 days preceding enrolment, or planned use during the study period
- Previous receipt of any recombinant adenoviral or recombinant MVA vectored vaccine
- Administration of immunoglobulins and/or any blood products within the three months preceding the planned administration of the vaccine candidate
- Any confirmed or suspected immunosuppressive or immunodeficient state, including HIV infection; asplenia; recurrent, severe infections and chronic (more than 14 days) immunosuppressant medication within the past 6 months (inhaled/topical steroids are allowed)
- History of allergic disease or reactions likely to be exacerbated by any component of the vaccine
- Any history of anaphylaxis in reaction to vaccination
- History of cancer (except basal cell carcinoma and cervical carcinoma in situ)

- History of serious psychiatric condition

- Any chronic illness requiring on-going or awaiting hospital specialist supervision, other than minor surgical procedures and follow up of surgery over 6 months prior to screening

- Suspected or known current injecting drug or alcohol abuse (as defined by an alcohol intake of greater than 42 units every week)

- Seropositive for hepatitis B surface (HBsAg) or hepatitis C virus (antibodies to HCV)

- Pregnancy, lactation or willingness/intention to become pregnant during the study

- Any other significant disease, disorder or finding (including blood test results), which, in the opinion of the Investigators, would either put the volunteer at risk because of participation in the study, or may influence the result of the study

- No response / confirmation from GP regarding previous medical history

Inclusion Criteria for Groups 5-6

The volunteer must satisfy all the following criteria to be eligible for the study:

- Healthy adults aged 50 or over, no upper age limit

- Able and willing (in the Investigator's opinion) to comply with all study requirements

- Willing to allow the investigators to discuss the volunteer's medical history with their General Practitioner

- For women of child bearing potential only, willingness to practice continuous effective contraception (i.e. hormonal contraception, intrauterine device or barrier contraception) during the study and a negative urinary pregnancy test on the day(s) of vaccination

- Agreement to refrain from blood donation during the course of the study

- Provide written informed consent

Exclusion Criteria for Groups 5-6

The volunteer may not enter the study if any of the following apply:

- Participation in another research study involving an investigational product in the 30 days preceding enrolment, or plans to participate during the study period

- Previous receipt of a vaccine or plans to receive any vaccinations during the study that would interfere with the interpretation of the results of the trial

- Administration of immunoglobulins and/or any blood products within the three months preceding the planned administration of the vaccine candidate

- Any confirmed or suspected immunosuppressive or immunodeficient state, including HIV infection; asplenia; recurrent, severe infections and chronic (more than 14 days) immunosuppressant medication within the past 6 months (inhaled/topical steroids including eye drops and nasal spray/intra-articular steroid injections are allowed)

- History of allergic disease or reactions likely to be exacerbated by any component of the vaccine including eggs or Kathon (a biocide added to body washes, conditioners, liquid soaps, shampoos and wipes as a preservative)

- Any history of anaphylaxis in reaction to vaccination

- History of treatment for cancer within the preceding six months (except basal cell carcinoma and cervical carcinoma in situ)

- History of serious psychiatric condition

- Any chronic illness requiring on-going or awaiting hospital specialist supervision, other than minor surgical procedures and follow up of surgery over 6 months prior to screening

- Suspected or known injecting drug abuse within the last 5 years

- Alcohol abuse (as defined by an alcohol intake of greater than 42 units every week)

- Seropositive for hepatitis B surface antigen (HBsAg) or hepatitis C virus (antibodies to HCV) or HIV

- Pregnancy, lactation or willingness/intention to become pregnant during the study

- Any other significant disease, disorder or finding (including blood test results), which, in the opinion of the Investigators, would either put the volunteer at risk because of participation in the study, or may influence the result of the study

- No response / confirmation from GP regarding previous medical history

6.4 Prevention of over-volunteering

Volunteers will be excluded from the study if they are concurrently involved in another trial. In order to check this, volunteers will be asked to provide their National Insurance or Passport number (if they are not entitled to a NI number) and will be registered on a national database of participants in clinical trials. (www.tops.org.uk).

6.5 Postponement Criteria

Vaccination will not proceed on the scheduled day in either of the following situations:

- The subject has a temperature > 37.5°C

- The Investigator judges the subject to have an acute moderate or severe illness (whether febrile or not)

All vaccines can be administered to persons with a minor illness, such as diarrhoea or mild upper respiratory infection, with or without low-grade fever, i.e., temperature of ≤37.5°C.

6.6 Re-vaccination exclusion criteria

The following adverse events associated with vaccine immunisation constitute absolute contraindications to further administration of vaccine. If any of these events occur during the study, the subject must be withdrawn and followed until resolution of the event, as with any adverse event.

- Anaphylactic reaction following administration of vaccine

- Pregnancy

6.7 Withdrawal Criteria

Subjects may be withdrawn from the study early:

- By withdrawing voluntarily
- On the decision of the Investigator
- On the advice of the local safety monitor (LSM)

The Investigator may withdraw the subject for any of the following reasons:

- Confirmed pregnancy during the trial
- Any adverse event which results in inability to comply with study procedures
- Ineligibility either arising during the study or retrospectively (having been overlooked at screening)
- Significant protocol deviation
- Subject non-compliance with study requirements
- Loss to follow up (applies to a subject who does not return for protocol study visits, is not reachable by telephone or other means of communication and/or is not able to be located).

The reason for withdrawal will be recorded in the CRF. Subjects withdrawn from the trial may be replaced at the discretion of the Investigators. If the subject is withdrawn due to an AE, the Investigator will arrange for appropriate specialist management or follow up visits or telephone calls until the AE has resolved or stabilised. The extent of follow up after premature discontinuation will be determined by the Investigator but will be at least for the whole study period, and if pregnant, until pregnancy outcome.

If a volunteer withdraws from the study, blood samples collected before their withdrawal from the trial will be used/ stored unless the volunteer specifically requests otherwise.

7. Treatment of Trial Volunteers

7.1 Study Procedures

Procedures will be performed on the visit time points indicated in the schedules of procedures (Tables 3-6). Additional procedures or laboratory tests may be performed at the discretion of the Investigators, e.g. an abnormal blood test may need repeating. However if a need for non-study related procedures is found (e.g. a chest X-ray) then this will be referred to the volunteer's General Practitioner.

7.2 Observations

Observations which will be documented are pulse, seated blood pressure and temperature. If any blood pressure result is significantly elevated, this will be repeated and the trend in results evaluated by a clinically trained member of the research team. Any persistent abnormalities will be referred to the volunteer's GP.

7.3 Blood Tests

Blood tests will be performed at clinic visits as documented in the schedules of procedures (Tables 3-6) and the following tests will be performed:

1. At the Oxford University Hospitals NHS Trust, University Hospital Southampton NHS Foundation Trust, St Helier Hospital and Surrey Pathology Services using NHS standard procedures:
    a. Haematology- Full blood count
    b. Biochemistry- Sodium, Potassium, Urea, Creatinine, Albumin, Liver Function Tests
    c. Diagnostic serology- HBsAg, HCV antibodies, HIV antibodies
    d. Immunology- Human Leucocyte Antigen (HLA) typing will be performed

2. At University of Oxford research laboratories:
    a. Exploratory immunology- including ELISpot and flow cytometry
    b. Gene expression studies

Some of this blood will be required for immediate use and the remainder stored indefinitely as a future research source, with the consent of the volunteers.

7.4 Urinalysis

Urine will be tested for blood, protein and glucose at screening. For female volunteers of child bearing potential, urine will be tested for beta-Human Chorionic Gonadotrophin (HCG) at screening and immediately prior to vaccination.

7.5 Contraception

All female volunteers of child bearing potential will be required to practice effective contraception for the duration of the trial. Methods of effective contraception include the oral contraceptive pill, a contraceptive hormonal implant, an intra-uterine device, condoms or a sterilisation procedure in the volunteer or their partner.

7.6 Vaccinations

Before each vaccination, the on-going eligibility of the volunteer will be reviewed. The vaccine will be administered as described in section 5.2. The injection site will be covered with a sterile dressing and the volunteer will stay in the clinical area for 1 hour (± 10 minutes) after vaccination, in case of immediate adverse events.

Observations will be taken 30 minutes (± 10 minutes) after vaccination and the sterile dressing removed and injection site inspected. The dressing will be discarded as GMO waste by autoclaving. An oral thermometer, tape measure and diary card will be given to each volunteer, with instructions on use. Volunteers will be provided with a 24 hour contact number so that they can speak to a member of the clinical team at any point.

7.7 Study visits

The study visits and procedures will be undertaken by one of the research team health professionals. The procedures to be included in each visit are documented in the schedules of attendances (Tables 3 to 6). Each visit is assigned a time point and a window period, within which the visit will be conducted.

Screening visit

All potential volunteers will have a screening visit, which may take place up to 90 days prior to vaccination. Informed consent will be taken as described in section 6.2. If consent is obtained, the screening procedures indicated in the schedule of procedures will be undertaken.

To avoid unnecessary additional venepuncture, if the appropriate blood test results for screening are available for the same volunteer from a screening visit for another study, these results may be used for assessing eligibility (provided the results date within the 90 days preceding enrolment in FLU005).

Abnormal clinical findings from the medical history, physical examination or blood tests at any point in the study will be assessed using the table in Appendix A or site-specific tables for Surrey volunteers. If a test is deemed clinically significant it may be repeated to ensure it is not a single occurrence. If an abnormal finding is deemed to be clinically significant, the volunteer will be informed and appropriate medical care arranged with the permission of the volunteer. Decisions to exclude the volunteer from the enrolling in the trial or to withdraw a volunteer from the trial will be at the discretion of the Investigator.

The eligibility of the volunteer will be reviewed at the end of the screening visit and again when all results from the screening visit have been considered.

Day 0- Enrolment and First Vaccination

Those volunteers eligible for enrolment, who wish to continue with the study, will be offered a day 0 visit to receive the first vaccine. Blood tests will be taken for Haematology, Biochemistry (as baseline), HLA type and gene expression studies. Volunteers will not be considered enrolled in the study until they have received a vaccine (Tables 3-6).

Groups 1-4

Safety Review – Day 1

Volunteers will be interviewed and assessed for local and systemic adverse events. Diary cards will be reviewed. Blood tests for gene expression studies will be taken. Temperature, blood pressure and pulse will be recorded.

A similar review will take place at week 8 + 1 day and week 52 + 1 day for volunteers receiving vaccinations on those dates. (Tables 3 and 4).

Follow up visits- weeks 1, 2, 3, 4, 8, (9, 10, 11) 26, 52, (53, 54, 55) 78

On these subsequent visits the volunteers will be assessed again for local and systemic adverse events. Once completed, the diary card will be collected at 1 week. Further history and physical examination will only be conducted if clinically indicated. Temperature, blood pressure and pulse will be recorded. Blood tests will be taken as detailed in the schedule of procedures (Tables 3 and 4).

Volunteers in groups 1 and 3 receive their second vaccination at week 8; they will need to attend follow-up at weeks 1, 2, 3, 4, 8, 9, 10, 11, 26 and once between weeks 52-55, as well as at week 78.

Volunteers in groups 2 and 4 receive their second vaccination at week 52; they will need to attend follow up at weeks 1, 2, 3, 4, once between weeks 8-11, as well as at weeks 26, 52, 53, 54, 55, and 78.

Groups 5 and 6

Safety Review – Day 2

Volunteers will be interviewed and assessed for local and systemic adverse events. Diary cards will be reviewed. Temperature, blood pressure and pulse will be recorded.

Follow up visits- Days 7, 14, 28, 56, (63, 84) 182

On these subsequent visits the volunteers will be assessed again for local and systemic adverse events. Once completed, the diary card will be collected at 1 week after each vaccination. Further history and physical examination will only be conducted if clinically indicated. Temperature, blood pressure and pulse will be recorded. Blood tests will be taken as detailed in the schedule of procedures (Tables 5 and 6).

Volunteers in group 5 receive a single vaccination at day 0; they will need to attend follow-up at weeks 1, 2, 4, 8 and 26.

For volunteers in group 6 receiving vaccination at day 0 and week 8, they will need to attend follow-up at weeks 1, 2, 4, 8, 9, 12 and 26.

	S	Vaccine 1						Vaccine 2							
Attendance number	1	2	3	4	5	6	7	8	9	10	11	12	13	14	15
Timeline	≥-90 days	0	Day 1	W1	W2	W3	W4	W8	W8+1	W9	W10	W11	W26	W52	W78
Window (days)		0	+1	±2	±2	±2	±2	±2	±1	±2	±2	±2	±7	±21	±14
Inclusion / Exclusion criteria	X	X						X							
Informed consent	X	(X)						(X)							
Medical History	X	(X)	(X)	(X)	(X)	(X)	(X)	(X)	(X)	(X)	(X)	(X)	(X)	(X)	(X)
Physical Examination	X	(X)	(X)	(X)	(X)	(X)	(X)	(X)	(X)	(X)	(X)	(X)	(X)	(X)	(X)
Urinalysis	X														
B-HCG urine test(♀)	X	X						X							
Review contraindications	X	X						X							
Vaccination		X						X							
Physical observations	X	X	X	X	X	X	X	X	X	X	X	X	(X)	(X)	(X)
AEs reviewed		X	X	X	X	X	X	X	X	X	X	X	X	X	X
Diary cards provided		X						X							
Diary cards collected				X						X					
HLA typing (mL)		4													
HBV,HCV,HIV (mL)	5														
Haematology (mL)	2	2		2	2			2		2	2		2		
Biochemistry (mL)	3	3		3	3			3		3	3		3		
Exploratory Immunology (mL)		60		60	60	60	60	60		60	60	60	60	60	60
PAXgene sample (mL)		2.5	2.5	2.5	2.5			2.5	2.5	2.5	2.5				
Blood volume per visit (mL)	10	71.5	2.5	67.5	67.5	60	60	67.5	2.5	67.5	67.5	60	65	60	60
Cumulative blood volume (mL)	10	81.5	84	151.5	219	279	339	406.5	409	476.5	544	604	669	729	789*

Table 3: *Schedule of attendances for groups 1 and 3, where group 1will receive ChAdOx1 NP+M1 2.5 x 10^{10} vp at Day 0 and MVA NP+M1 1.5 x 10^{8} pfu at week 8, and group 3 will receive MVA NP+M1 1.5 x 10^{8} pfu at day 0 and ChAdOx1 NP+M1 2.5 x 10^{10} vp at week 8.*

(Windows refer to time since last visit). S = screening visit, (x) = If considered necessary

** Cumulative blood volume for Oxford volunteers if blood taken as per schedule, and excluding any repeat safety blood test that may be necessary. Southampton volunteers may have a slightly higher cumulative volume due to use of higher volume vacutainers for biochemistry, haematology and serology samples as per local Trust standard procedures.*

	S	Vaccine 1								Vaccine 2					
Attendance number	1	2	3	4	5	6	7	8	9	10	11	12	13	14	15
Timeline	≥-90 days	0	Day 1	W1	W2	W3	W4	W8	W26	W52	W52+1	W53	W54	W55	W78
Window (days)		0	+1	±2	±2	±2	±2	±21	±7	±14	±1	±2	±2	±2	±7
Inclusion / Exclusion criteria	X	X													
Informed consent	X	(X)													
Medical History	X	(X)	(X)	(X)	(X)	(X)	(X)	(X)	(X)	(X)	(X)	(X)	(X)	(X)	(X)
Physical Examination	X	(X)	(X)	(X)	(X)	(X)	(X)	(X)	(X)	(X)	(X)	(X)	(X)	(X)	(X)
Urinalysis	X														
B-HCG urine test(♀)	X	X								X					
Review contraindications	X	X								X					
Vaccination		X								X					
Physical observations	X	X	X	X	X	X	X	(X)	(X)	X	X	X	X	X	(X)
AEs reviewed		X	X	X	X	X	X	X	X	X	X	X	X	X	X
Diary cards provided		X								X					
Diary cards collected				X								X			
HLA typing (mL)		4													
HBV,HCV,HIV (mL)	5														
Haematology (mL)	2	2		2	2			2		2		2	2		
Biochemistry (mL)	3	3		3	3			3		3		3	3		
Exploratory Immunology (mL)		60		60	60	60	60	60	60	60		60	60	60	60
PAXgene sample (mL)		2.5	2.5	2.5	2.5					2.5	2.5	2.5	2.5		
Blood volume per visit (mL)	10	71.5	2.5	67.5	67.5	60	60	65	60	67.5	2.5	67.5	67.5	60	60
Cumulative blood volume (mL)	10	81.5	84	151.5	219	279	339	404	464	531.5	534	601.5	669	729	789*

Table 4: *Schedule of attendances for groups 2 and 4, where group 2 will receive ChAdOx1 NP+M1 2.5 x 10^{10} vp at Day 0 and MVA NP+M1 1.5 x 10^{8} pfu at week 52, and group 4 will receive MVA NP+M1 1.5 x 10^{8} pfu at day 0 and ChAdOx1 NP+M1 2.5 x 10^{10} vp at week 52.*

(Windows refer to time since last visit). S = screening visit, (x) = If considered necessary

** Cumulative blood volume for Oxford volunteers if blood taken as per schedule, and excluding any repeat safety blood test that may be necessary. Southampton volunteers may have a slightly higher cumulative volume due to use of higher volume vacutainers for biochemistry, haematology and serology samples as per local Trust standard procedures.*

	S	Vaccine 1						
Attendance number	1	2	3	4	5	6	7	8
Timeline (days)	≥-90 days	0	D2	D7	D14	D28	D56	D182
Window (days)		0	±1	±2	±2	±2	±2	±7
Inclusion / Exclusion criteria	X	X						
Informed consent	X	(X)						
Medical History	X	(X)	(X)	(X)	(X)	(X)	(X)	(X)
Physical Examination	X	(X)	(X)	(X)	(X)	(X)	(X)	(X)
Urinalysis	X							
B-HCG urine test(♀)	X	X						
Review contraindications	X	X						
Vaccination		X						
Physical observations	X	X	X	X	X	X	X	(X)
AEs reviewed		X	X	X	X	X	X	X
Diary cards provided		X						
Diary cards collected				X				
HLA typing (mL)		4						
HBV,HCV,HIV (mL)	4							
Haematology (mL)	2	2		2		2		
Biochemistry (mL)	2.5	2.5		2.5		2.5		
Exploratory Immunology (mL)		60		60	60	60	60	60
Blood volume per visit (mL)	8.5	68.5	0	64.5	60	64.5	60	60
Cumulative blood volume (mL)	8.5	77	77	141.5	201.5	266	326	386*

Table 5: *Schedule of attendances for group 5, receiving ChAdOx1 NP+M1 2.5 x 10^{10} vp at Day 0 (Windows refer to time since last visit).* S = screening visit, (x) = If considered necessary

**Cumulative blood volume for Surrey volunteers if blood taken as per schedule, and excluding any repeat safety blood test that may be necessary.*

	S	Vaccine 1					Vaccine 2			
Attendance number	1	2	3	4	5	6	7	8	9	10
Timeline (days)	≥-90 days	0	D2	D7	D14	D28	D56	D63	D84	D182
Window (days)		0	±1	±2	±2	±2	±2	±2	±2	±7
Inclusion / Exclusion criteria	X	X					X			
Informed consent	X	(X)					(X)			
Medical History	X	(X)	(X)	(X)	(X)	(X)	(X)	(X)	(X)	(X)
Physical Examination	X	(X)	(X)	(X)	(X)	(X)	(X)	(X)	(X)	(X)
Urinalysis	X									
B-HCG urine test(♀)	X	X					X			
Review contraindications	X	X					X			
Vaccination		X					X			
Physical observations	X	X	X	X	X	X	X	X	X	(X)
AEs reviewed		X	X	X	X	X	X	X	X	X
Diary cards provided		X					X			
Diary cards collected				X				X		
HLA typing (mL)		4								
HBV,HCV,HIV (mL)	4									
Haematology (mL)	2	2		2		2	2	2	2	
Biochemistry (mL)	2.5	2.5		2.5		2.5	2.5	2.5	2.5	
Exploratory Immunology (mL)		60		60	60	60	60	60	60	60
Blood volume per visit (mL)	8.5	68.5	0	64.5	60	64.5	64.5	64.5	64.5	60
Cumulative blood volume (mL)	8.5	77	77	141.5	201.5	266	330.5	395	459.5	519.5*

Table 6: *Schedule of attendances for group 6, receiving ChAdOx1 NP+M1 2.5 x 10^{10} vp at Day 0 and MVA NP+M1 1.5 x 10^{8} pfu at week 8. (Windows refer to time since last visit).* S = screening visit, (x) = If considered necessary

**Cumulative blood volume for Surrey volunteers if blood taken as per schedule, and excluding any repeat safety blood test that may be necessary.*

8. Assessment of Safety

Safety will be assessed by the nature, frequency, severity, seriousness and duration of adverse events arising during the study.

8.1 Definitions

Adverse Event (AE)

An AE is any untoward medical occurrence in a volunteer, including a dosing error, which may occur during or after study vaccination and does not necessarily, have to have a causal relationship with vaccination. An AE can therefore be any unfavourable and unintended sign (including an abnormal laboratory finding), symptom or disease temporally associated with study vaccination, whether or not considered related to study vaccination.

Adverse Reaction (AR)

An AR is any untoward or unintended response to a study vaccination. This means that a causal relationship between the study vaccination and an AE is at least a reasonable possibility, i.e., the relationship cannot be ruled out. All cases judged by either the reporting medical investigator or the sponsors as having a reasonable suspected causal relationship to a study vaccination (i.e. possibly, probably or definitely related to a study vaccination) will qualify as adverse reactions.

Unexpected Adverse Reaction

An unexpected adverse reaction is where the nature or severity is not consistent with the Investigator's Brochure.

Serious Adverse Event (SAE)

To ensure no confusion or misunderstanding of the difference between the terms "serious" and "severe", which are not synonymous, the following note of clarification is provided: The term "severe" is often used to describe the intensity (severity) of a specific event (as in mild, moderate, or severe myocardial infarction); the event itself, however, may be of relatively minor medical significance (such as severe headache). This is not the same as "serious," which is based on patient/event outcome or action criteria usually associated with events that pose a threat to a volunteer's life or functioning. Seriousness (not severity) serves as a guide for defining regulatory reporting obligations.

An SAE is an AE that results in any of the following outcomes, whether or not considered related to the study intervention.

- Death (i.e., results in death from any cause at any time)
- Life-threatening event (i.e., the volunteer was, in the view of the investigator, at immediate risk of death from the event that occurred). This does not include an AE that, if it occurred in a more serious form, might have caused death.
- Persistent or significant disability or incapacity (i.e. substantial disruption of one's ability to carry out normal life functions).
- Hospitalisation, regardless of length of stay, even if it is a precautionary measure for continued observation. Hospitalisation (including inpatient or outpatient hospitalization for an elective procedure) for a pre-existing condition that has not worsened unexpectedly does not constitute a serious AE.

- An important medical event (that may not cause death, be life threatening, or require hospitalization) that may, based upon appropriate medical judgment, jeopardize the volunteer and/or require medical or surgical intervention to prevent one of the outcomes listed above. Examples of such medical events include allergic reaction requiring intensive treatment in an emergency room or clinic, blood dyscrasias, or convulsions that do not result in inpatient hospitalization.
- Congenital anomaly or birth defect.

Serious Adverse Reaction (SAR)

An adverse event (expected or unexpected) that is both serious and, in the opinion of the reporting investigator or sponsors, believed to be possibly, probably or definitely due to a study vaccination or any other study treatments, based on the information provided.

Suspected Unexpected Serious Adverse Reaction (SUSAR)

A SUSAR is different from an SAE in that it is unexpected and thought to be related to the investigational product. Reports of any SUSAR will be sent to the MHRA and REC within 7 days for fatal and life-threatening cases and within 15 days for all other SUSARs. Administration of further vaccines within the trial will be suspended until a safety review is convened.

Foreseeable Adverse Reactions

The foreseeable ARs following vaccination with ChAdOx1 NP+M1 and MVA NP+M1 include injection site pain, erythema, warmth, swelling, pruritus, myalgia, arthralgia, headache, fatigue, fever, feverishness, malaise and nausea.

8.2 Causality Assessment

For every AE, an assessment of the relationship of the event to the administration of the vaccine will be undertaken. An intervention-related AE refers to an AE for which there is a probable or definite relationship to administration of a vaccine. An interpretation of the causal relationship of the intervention to the AE in question will be made, based on the type of event; the relationship of the event to the time of vaccine administration; and the known biology of the vaccine therapy (Table 7).

0	**No Relationship**	No temporal relationship to study product ***and***<br>Alternate aetiology (clinical state, environmental or other interventions); ***and***<br>Does not follow known pattern of response to study product
1	**Unlikely**	Unlikely temporal relationship to study product ***and***<br>Alternate aetiology likely (clinical state, environmental or other interventions) ***and***<br>Does not follow known typical or plausible pattern of response to study product.
2	**Possible**	Reasonable temporal relationship to study product; ***or***<br>Event not readily produced by clinical state, environmental or other interventions; ***or***<br>Similar pattern of response to that seen with other vaccines
3	**Probable**	Reasonable temporal relationship to study product; ***and***<br>Event not readily produced by clinical state, environment, or other interventions ***or***<br>Known pattern of response seen with other vaccines
4	**Definite**	Reasonable temporal relationship to study product; ***and***<br>Event not readily produced by clinical state, environment, or other interventions; ***and***<br>Known pattern of response seen with other vaccines

Table 7: *Guidelines for assessing the relationship of vaccine administration to an AE*

8.3 Severity Assessment

The severity of adverse events will be assessed according to the scales in Tables 8 and 9.

Grade	Description	Definition
**0**		Absence of the indicated symptom
**1**	Mild	Awareness of a symptom but the symptom is easily tolerated
**2**	Moderate	Discomfort enough to cause interference with usual activity
**3**	Severe	Incapacitating; unable to perform usual activities; requires absenteeism or bed rest

Table 8: *Generic scale for assessing the severity of AEs*

Volunteers will be asked to record their temperature on a daily basis for one week post each vaccination, as well as the size of any swelling or redness at the vaccination site. Severity of pain at the injection site will also be assessed daily. For these solicited AEs (with a numerical value) severity will be assigned according to the following scales:

Adverse Event	Grade	Intensity
Erythema at injection site*	1	>3 - ≤50 mm
	2	>50 - ≤100 mm
	3	>100 mm
Swelling at injection site	1	>1 - ≤20 mm
	2	>20 - ≤50 mm
	3	>50 mm
Fever (oral)	1	37.6°C - 38.0°C
	2	38.1°C – 39.0°C
	3	>39.0°C
Pain at injection site	1	Pain that is easily tolerated
	2	Pain that interferes with daily activity
	3	Pain that prevents daily activity

Table 9: *Scale for assessing the severity of solicited AEs with numerical values; * erythema ≤3mm is an expected consequence of skin puncture and will therefore not be considered an adverse event*

The severity of laboratory test abnormalities will be assessed according to site-specific tables stored in the TMF based on the local reference ranges.

8.4 Procedures to be followed in the event of abnormal findings

Abnormal clinical findings from medical history, examination or blood tests, will be assessed for their clinical significance. If a test is deemed clinically significant, it may be repeated, to ensure it is not a single occurrence. If a test remains clinically significant, the volunteer will be informed and appropriate medical care arranged as appropriate and with the permission of the volunteer. Decisions to exclude the volunteer from the enrolling in the trial or to withdraw a volunteer from the trial will be at the discretion of the Investigator.

8.5 Reporting Procedures for all Adverse Events

All AEs occurring during the study observed by the Investigators or reported by the volunteer, whether or not attributed to vaccination, will be reported in the CRF. All AEs that result in a patient's withdrawal from the study will be followed up until a satisfactory resolution occurs, or until a non-study related causality is assigned. All deaths occurring during the study will be reported to the Sponsor. For all deaths, available autopsy reports and relevant medical reports will be made available for reporting to the relevant authorities.

Reporting Procedures for Serious Adverse Events

All SAEs will be documented accurately and notification deadlines respected as specified in the site specific SOP (e.g. CCVTM SOP OVC005). SAEs will be reported within 24 hours by the Investigator to the Sponsor or person with appropriate responsibility delegated from the Sponsor (Chief Investigator), and to the Local Safety Monitor. This will be performed by emailing an electronic version of the completed SAE Initial Report Form (CCVTM form VC004F1). Any relevant information concerning the SAE that becomes available after the SAE Initial Report Form has been sent (outcome, precise history, results of investigations, copy of hospital report, etc) will be forwarded in a timely manner using the SAE Update Report Form (CCVTM form VC004F1). The Sponsor, or person with appropriate responsibility delegated from the Sponsor (medically qualified investigator), is responsible for commencing, maintaining and completing the SAE Sponsor Report Form (VC004F1). The anonymity of subjects shall be respected when forwarding this information.

In addition to the expedited reporting requirements, the Investigator shall submit a Development Safety Update Report (DSUR) to the ethical committee and MHRA (and a copy to the Sponsor) on the anniversary of the first Clinical Trial Application (CTA) for FLU005/Development International Birth Date (DIBD).

Reporting Procedures for SUSARs

If the SAE is a SUSAR it will be reported to the ethical committee and MHRA within 7 days of the Chief Investigator having first knowledge of the event (for fatal and life-threatening cases) and within 15 days for all other SUSARs (and the report copied to the Sponsor). For all deaths, available autopsy reports will be made available for reporting to the regulatory authorities. The Chief Investigator will also inform all investigators concerned of relevant information about SUSARs that could adversely affect the safety of participants.

SAEs which are not SUSARs will not normally be reported to the ethical committee and MHRA, unless there is a clinically important increase in occurrence rate, an unexpected outcome, or a new event that is likely to affect safety of trial volunteers.

8.6 Local Safety Monitor

A Local Safety Monitor (LSM) or their delegate will be available to provide safety oversight. The LSM will be informed of all SAEs by email. The LSM or his delegate will be notified within 24 hours of the Investigators' being aware of their occurrence. The LSM has the power to terminate the study if deemed necessary following a vaccine-related SAE. At the time of writing the LSM is Dr Brian Angus, Clinical Tutor in Medicine, Honorary Consultant Physician and Director of the Centre for Tropical Medicine at Oxford University.

8.7 Pregnancy

Subjects who become pregnant during the study after receiving the vaccine may continue other study procedures at the discretion of the Investigator. The Investigator will collect pregnancy information on any subject who becomes pregnant while participating in this study. The volunteer will be followed to determine the outcome of the pregnancy.

9. Statistics

Safety data will be presented according to frequency, severity and duration of adverse events.

The primary analysis will involve calculating the area under the curve of the immune response (IFN-γ SFC/ million PBMCs) for each volunteer from baseline to week 78 for groups 1-4, or week 26 for groups 5 and 6. The mean area under the curve in groups 1-4 and 5-6 will then be compared using an analysis of variance, or in the case of skewed data a Kruskal Wallis test. Further pairwise comparisons will be made if a significant difference is found between groups.

The secondary outcomes of peak immune response after first vaccination and peak immune response after second vaccination will be analysed in the same way.

Power Calculations

In this study there will initially be four groups of 12 subjects each. This gives 88% power to see a mean three-fold increase from pre-existing T cell response to peak post-vaccination response in response to NP and M1 at the 5% significance level. Since the trial will run for eighteen months, it is possible that there could be some drop out of subjects. With 10 per group, we will still have 80%, and at a worst case scenario of 8 per group we would have 68% power.

For the older volunteers in groups 5 and 6 we will also recruit 12 subjects per group. This is an exploratory study as the extremely high variability of T cell response to influenza and influenza vaccines in older volunteers means that we currently do not have enough data on which to base power calculations.

10. Serious Breaches

The Medicines for Human Use (Clinical Trials) Regulations contain a requirement for the notification of "serious breaches" to the MHRA within 7 days of the sponsor becoming aware of the breach.

A serious breach is defined as “A breach of GCP or the trial protocol which is likely to affect to a significant degree:

(a) The safety or physical or mental integrity of the subjects of the trial; or

(b) The scientific value of the trial”.

In the event that a serious breach is suspected the sponsor will be informed within one working day.

11. Quality Control and Quality Assurance Procedures

Data will be evaluated for compliance with the protocol and accuracy in relation to source documents. The study will be conducted in accordance with procedures identified in the protocol. SOPs will be used at all clinical and laboratory sites. Regular monitoring will be performed according to ICH Good Clinical Practice (GCP). Following written standard operating procedures, the monitors will verify that the clinical trial is conducted and data are generated, documented and reported in compliance with the protocol, GCP and the applicable regulatory requirements. The investigator site will provide direct access to all trial related source data/documents and reports for the purpose of monitoring and auditing by the sponsor, and inspection by local and regulatory authorities.

12. Ethics

12.1 Good Clinical Practice (GCP)

This trial will be conducted in accordance with the principles of the Declaration of Helsinki as agreed by the World Medical Association General Assembly (Washington 2002), ICH Good Clinical Practice (GCP), the requirements of the Medicines for Human Use (Clinical Trial) Regulations 2004, and local regulatory requirements.

12.2 Ethical Review

A copy of the protocol, proposed informed consent form, other written volunteer information and the proposed advertising material will be submitted to an independent EC for written approval. The Investigators will submit and, where necessary, obtain approval from the EC for all subsequent substantial amendments to the protocol and informed consent document (and previously approved by the sponsor). The Investigators will notify serious deviations from the protocol or SAEs occurring at the site to the Sponsor and will notify the EC of these in accordance with local procedures.

12.3 Informed Consent

Written informed consent will be obtained at screening as detailed in section 6.2.

13. Data Handling and Record Keeping

13.1 Data Handling

The Principal Investigator will have overall responsibility for receiving, entering, cleaning, querying, analysing and storing all data that accrues from the study, but these tasks may be delegated to other Investigators. Data will be entered into the volunteers' CRFs in a paper and/or electronic format (using the OpenClinica™ database). Electronic data will be stored on secure servers which are outsourced by OpenClinica™.

This includes safety data, laboratory data (both clinical and immunological) and outcome data. Data is entered in a web browser on PCs and then transferred to the OpenClinica Database by encrypted (Https) transfer.

13.2 Record Keeping

The Investigators will maintain appropriate medical and research records for this trial, in compliance with the requirements of the Medicines for Human Use (Clinical Trial) Regulations 2004, ICH E6 GCP and regulatory and institutional requirements for the protection of confidentiality of volunteers. The Chief Investigator, Principal Investigator, co-investigators and clinical research nurses will have access to records. The Investigators will permit authorized representatives of the sponsor, and regulatory agencies to examine (and when required by applicable law, to copy) clinical records for the purposes of quality assurance reviews, audits and evaluation of the study safety and progress.

13.3 Source Data and Case Report Forms (CRFs)

All protocol required information will be collected in CRFs designed by the Investigators. All source documents will be filed in the CRF. Source documents are original documents, data, and records from which the volunteer's CRF data are obtained. For this study, these will include, but are not limited to, volunteer consent form, blood results, GP response letters, laboratory records and diaries. In the majority of cases, CRF entries will be considered source data as the CRF is the site of the original recording (i.e., there is no other written record of data). In this study this will include, but is not limited to medical history, medication records, vital signs, physical examination records, urine assessments, blood results, adverse event data and details of vaccinations. All source data and volunteer CRFs will be stored securely and anonymised prior to archiving.

13.4 Data Protection

The study protocol, documentation, data and all other information generated will be held in strict confidence. No information concerning the study or the data will be released to any unauthorized third party, without prior written approval of the sponsor.

14. Financing and Insurance

14.1 Financing

This study will be funded by a grant for the Medical Research Council (MRC)

14.2 Insurance

Negligent Harm

The University has arrangements in place to provide for harm arising from participation in the study for which the University is the Research Sponsor.

Non-Negligent Harm

The University has arrangements in place to provide for non-negligent harm arising from participation in the study for which the University is the Research Sponsor.

14.3 Compensation

Subjects will be compensated for their time and for the inconvenience caused by procedures. The amount of compensation will be stated in the volunteer information sheet. If subjects withdraw without completing the study then they are entitled to receive a pro-rata amount of compensation. If volunteers need to have additional visits (e.g. for repeats of safety bloods) then additional compensation can be given. Where volunteers incur higher travel costs due to additional unanticipated visits then appropriate compensation will provided.

GPs requesting payment for returning the response letter and sending an invoice will receive remuneration.

15. References

1. Lagace-Wiens, P.R., E. Rubinstein, and A. Gumel, *Influenza epidemiology--past, present, and future.* Crit Care Med, 2010. **38**(4 Suppl): p. e1-9.
2. Treanor, J., *Influenza Viruses, Including Avian Influenza and Swine Influenza.* Principles and Practice of Infectious Diseases. Mandell GL, Bennett JE, Dolin R (Eds). **2**: p. 2060–2085.
3. Molinari, N.A., et al., *The annual impact of seasonal influenza in the US: measuring disease burden and costs.* Vaccine, 2007. **25**(27): p. 5086-96.
4. Babakir-Mina, M., et al., *Influenza virus A (H5N1): a pandemic risk?* New Microbiol, 2007. **30**(2): p. 65-78.
5. Kilbourne, E.D., et al., *The total influenza vaccine failure of 1947 revisited: major intrasubtypic antigenic change can explain failure of vaccine in a post-World War II epidemic.* Proc Natl Acad Sci U S A, 2002. **99**(16): p. 10748-52.
6. Hannoun, C., F. Megas, and J. Piercy, *Immunogenicity and protective efficacy of influenza vaccination.* Virus Res, 2004. **103**(1-2): p. 133-8.
7. Bardenheier, B.H., et al., *Influenza vaccine supply, 2005-2006: did we come up short?* BMC Health Serv Res, 2007. **7**: p. 66.
8. Berthoud, T.K., et al., *Potent CD8+ T-cell immunogenicity in humans of a novel heterosubtypic influenza A vaccine, MVA-NP+M1.* Clin Infect Dis, 2011. **52**(1): p. 1-7.
9. Bett, A.J., et al., *An efficient and flexible system for construction of adenovirus vectors with insertions or deletions in early regions 1 and 3.* Proc Natl Acad Sci U S A, 1994. **91**(19): p. 8802-6.
10. Top, F.H., Jr., et al., *Immunization with live types 7 and 4 adenovirus vaccines. II. Antibody response and protective effect against acute respiratory disease due to adenovirus type 7.* J Infect Dis, 1971. **124**(2): p. 155-60.
11. Dicks, M.D., et al., *A novel chimpanzee adenovirus vector with low human seroprevalence: improved systems for vector derivation and comparative immunogenicity.* PLoS One, 2012. **7**(7): p. e40385.
12. O'Hara, G.A., et al., *Clinical assessment of a recombinant simian adenovirus ChAd63: a potent new vaccine vector.* J Infect Dis, 2012. **205**(5): p. 772-81.
13. Lillie, P.J., et al., *Preliminary assessment of the efficacy of a T-cell-based influenza vaccine, MVA-NP+M1, in humans.* Clin Infect Dis, 2012. **55**(1): p. 19-25.
14. FDA, *Toxicity Grading Scale for Healthy Adult & Adolescent Volunteers Enrolled in Preventative Vaccine Clinical Trials.*

Appendix A. Laboratory values for exclusion

The following reference ranges are provided for the purpose of guidance only for Investigators to assess eligibility for the trial. Results that fall outside these ranges may not be of clinical significance but should be considered on an individual basis. The below values are based upon the reference ranges used by the laboratories in Oxford.

Blood tests undertaken at all sites should refer to the site-specific tables for grading of severity of laboratory adverse events. **In Surrey**, all results that would be classified as a Grade 1 abnormality or higher should be considered on an individual basis**.**

**PARAMETER**	**LOWER LIMIT**<br>**OF EXCLUSION**	**UPPER LIMIT**<br>**OF EXCLUSION**
**BIOCHEMISTRY**		
Potassium [mmol/L]	<3.2	>5.5
Sodium [mmol/L]	<132	>148
Urea [mmol/L]	N/A	>9
Creatinine [μmol/L]	N/A	>145
Albumin [g/L]	<30	N/A
Total bilirubin [μmol/L]	N/A	>19 when accompanied by elevated liver enzyme
ALT [IU/L]	N/A	>60
ALP [IU/L]	N/A	>350
**HAEMATOLOGY**		
Haemoglobin [g/dL]	Male: < 11.5<br>Female: < 10.5	Male: > 18<br>Female: >17.5
White Cell Count [x 10^9/L]<br>Neutrophil count [x 10^9/L]	<3.5<br>< 1.5	>14.0
Platelet Count [x 10^9/L]	<130	>500
**URINE ANALYSIS (using MULTISTIX * 10 SG Bayer Diagnostics)**		
Protein [g/L]	> 0.3	
Glucose [mmol/L]	> 5.5	
