# Supplementary material for: Heterologous Two-Dose Vaccination with Simian Adenovirus and Poxvirus Vectors Elicits Long-Lasting Cellular Immunity to Influenza Virus A in Healthy Adults
Source: eBioMedicine. 2018 Feb 15;29:146–54. doi: 10.1016/j.ebiom.2018.02.011 (PMC5926543; doi:10.1016/j.ebiom.2018.02.011)
Supplement: Supplementary file 2 — Supplementary material 2 [file mmc2.docx]

**Supplementary figures, table and methods**

Supplementary Fig. 1: Comparison of baseline IFN-γ ELISpot responses between G1-6. No significant differences were observed in the IFN-γ ELISpot responses to vaccine antigen NP+M1 at baseline (W0).

*

Supplementary Fig. 2: Area under the curve (AUC) analysis. An MVA-NP+M1 boost at W8 in older adults (G6) improves the overall immune response when compared with an initial vaccination with ChAdOx1 NP+M1 alone (G5) in the interval W0-W26. Abbreviations are as follows; AUC = area under the curve, ChAd = ChAdOx1 NP+M1 and MVA = MVA-NP+M1. Differences were calculated using an unpaired non-parametric test (Mann-Whitney),* = p<0·05.

**G1&G2: ChAd-MVA**

**G3&4: MVA-ChAd**

**G5&6: ChAd-MVA**

Supplementary Fig. 3: Breadth of the IFN-γ ELISpot response. Radar charts comparing the breadth of the immune response to NPM1 peptide pools P1-8 following vaccination with ChAdOx1 NP+M1 and/or MVA-NP+M1 at W0 or W78. The values on the axis represent the proportion of the response in each pool (P1-8) as a percentage of the total response. The proportion of pre-vaccination (W0), and at the end of the study (W78; G1-4 or W26; G5&6) responses for each group are shown.

**G5&6: ChAd-MVA**

Supplementary Fig. 4: Gating strategy for flow cytometry. PBMC were gated on lymphocytes, singlets and CD3+ live cells with a negative dump channel for CD14/CD19/CD56-positive populations. CD4+ and CD8+ T-cells were identified and individual cytokine negative and positive populations gated (e.g. IFN-γ, CD107α or TNF-α). Unstimulated cells (UNST) and stimulated cells (SEB) for IFN-γ only are shown and were used to identify negative and positive populations. Identical gates were applied for each volunteer within a single group at each time-point.

Supplementary Fig. 5. Gating of T-cell memory markers. PBMC responses to stimulation with vaccine antigen P1-8 or SEB were assessed. An identical gating approach was used as in Supplementary Fig.4 with an additional gate for CD8+ CCR7/CD45RA. This gate was applied to IFN-γ+ producing CD8+ T-cells to determine their memory phenotype (for UNST, P1-8 and SEB). TCM = CCR7+/CD45RA-, TEM = CCR7-/CD45RA-, TEMRA = CCR7-/CD45RA+ or naive = CCR7+/CD45RA+. Surface receptor and intracellular staining was analyzed using FlowJo software (version.10).

Supplementary Fig. 6: Phenotyping IAV-specific IFN-γ+ CD8+ T-cells. The phenotype of antigen specific T-cell responses to pooled peptides from the NP+M1 vaccine antigen were analyzed by expression of memory markers CD45RA/CCR7 for G1-4 (A, B) and G5&6 (C, D). The gating strategy is outlined in Supplementary Fig.4& 5. IFN-γ+ CD8+ T-cell responses following 1st vaccination (V1), peak of response post-vaccination (V1+7), second vaccination (V2), peak response following vaccination (V2+7) and W26 or W78 were analyzed in frozen PBMCs. Abbreviations are as follows; TCM = central memory T-cells (CCR7+/CD45RA-) and TEM = effector memory (CCR7-/CD45RA-). Values shown are mean -/+ SEM.

a

b

c

d

Supplementary Table 1: Antibody panel for flow cytometry

| Marker | Conjugate | Antibody Clone | Volume/test (µl)* |
| --- | --- | --- | --- |
| IFY-γ | FITC | 4S.B3 | 0.2 |
| IL-2 | PE | MQ1-17H12 | 1.0 |
| CD107α | PE-Cy5 | eBioH4A3 | 4.0 |
| CD14 | eFluor450 | 61D3 | 0.1 |
| CD19 | eFluor450 | H1B19 | 1.0 |
| CD56 | eFluor450 | HCD56 | 1.0 |
| LIVE/DEAD | Fixable Aqua | N/A | 5.0 |
| CD4 | APC | RPA-T4 | 2.0 |
| CD3 | AF700 | UCHT1 | 1.0 |
| CD8 | APC-dFluor780 | RPA-T8 | 5.0 |
| CCR7 | PerCP-Cy5.5 | 150503 | 2.5 |
| CD45RA | QD655 | MEM56 | 0.5 |

*****2 x 10^6^ cells analyzed per test

**SUPPLEMENTARY METHODS**

**Breadth of the IFN-γ ELISpot response**

Values for individual peptide pools spanning NP+M1 were separated into eight separate pools (P1-8). Boosting of pre-existing responses within pools following each vaccination are expressed as a proportion of the total summed response (Supplementary Fig.3).

**Intracellular Cytokine Staining (ICS) and Analysis by Flow Cytometry**

Cryopreserved PBMCs were thawed and treated with 2μl/ml of 25U/ml benzonase (Merck Chemicals Ltd) for 2h, washed, centrifuged and resuspended in R10. Two million PBMCs were incubated with 1μg/ml of co-stimulatory antibodies αCD28 and αCD49d (BD Pharmingen) and anti-CD107a followed by stimulation in polypropylene FACS tubes with 1) R10 alone (negative control), 2) Staphylococcal Enterotoxin B (positive control; 1μg/ml) or 3) a single pool of all 80 NP+M1 peptides at a final concentration of 4μg/ml (P1-8). Following 2h of incubation, 10 μg/ml brefeldin A and monensin (eBiosciences) was added to each sample and cells incubated for a further 16-18 hours at 37°C. PBMCs were stained with an 11-colour panel of antibodies (for details see Supplementary Table.1). Dead cells were discriminated by live/dead fixable aqua staining (Invitrogen for 20min at room temperature). Cells were washed with FACS buffer (PBS containing 1% bovine serum albumin and 0·01% sodium azide) and incubated for 20min with Cytofix/Cytoperm (BD Biosciences). Cells were washed with Perm Wash buffer (BD Biosciences), pelleted by centrifugation and incubated with the antibody cocktail for 30min at room temperature. Cells were fixed in 1% paraformaldehyde and acquired on the same day as staining. Monocytes (CD14^+^), B-cells (CD19^+^) and NK cells (CD56^+^) were excluded from analysis. Cells were gated on lymphocytes, singlets, live cells, CD3^+^CD14^-^CD19^-^, CD4^+^CD8^-^ or CD8^+^CD4^-^, then assessed for IFN-γ, IL-2, TNFα secretion, combinations of these cytokines and for CD107a expression. A sample gating strategy is provided in Supplementary Fig. 4.

Over 500,000 live gated lymphocyte events were acquired on a Beckton Dickinson LSRII flow cytometer using FACSDiva software (BD Biosciences) and data analyzed using Flow Jo, Version 10 (Tree Star Inc). OneComp ebeads (Affymetrix Biosciences) were used to automatically calculate the relevant single fluorochrome compensation controls and photo multiplier tube voltages were set by daily acquisition of Cytometer Setup and Tracking beads (BD Biosciences). All antibodies were titrated for optimal staining.

Responses to peptide were determined by subtraction of the unstimulated control for each sample. A cytokine response is deemed positive if greater than the background response, is representative of >50 cells and is above the lower limit of detection for CD4^+^ and CD8^+^ T cells (calculated by [1/lowest number of CD4^+^ or CD8^+^ cells acquired in antigen-stimulated or unstimulated samples]x100). A sample passed positive QC if cytokine production for at least 1 cytokine was >1% in SEB stimulated cells.
